# Analyses of plasma metabolites using a high performance four-channel CIL LC-MS method and identification of metabolites associated with enteric methane emissions in beef cattle

**DOI:** 10.1371/journal.pone.0299268

**Published:** 2024-03-01

**Authors:** Hongwei Li, Xiaohang Wang, Michael Vinsky, Ghader Manafiazar, Carolyn Fitzsimmons, Liang Li, Changxi Li

**Affiliations:** 1 Department of Agricultural, Food and Nutritional Science, University of Alberta, Edmonton, Alberta, Canada; 2 Department of Chemistry, University of Alberta, Edmonton, Alberta, Canada; 3 Lacombe Research and Development Centre, Agriculture and Agri-Food Canada, Lacombe, Alberta, Canada; 4 Department of Animal Science and Aquaculture, Faculty of Agriculture, Dalhousie University, Truro, Nova Scotia, Canada; West Virginia University, UNITED STATES

## Abstract

Reducing enteric methane (one greenhouse gas) emissions from beef cattle not only can be beneficial in reducing global warming, but also improve efficiency of nutrient utilization in the production system. However, direct measurement of enteric methane emissions on individual cattle is difficult and expensive. The objective of this study was to detect plasma metabolites that are associated with enteric methane emissions in beef cattle. Average enteric methane emissions (CH_4_) per day (AVG_DAILYCH4) for each individual cattle were measured using the GreenFeed emission monitoring (GEM) unit system, and beef cattle with divergent AVG_DAILYCH4 from Angus (n = 10 for the low CH_4_ group and 9 for the high CH_4_ group), Charolais (n = 10 for low and 10 for = high), and Kinsella Composite (n = 10 for low and 10 for high) populations were used for plasma metabolite quantification and metabolite-CH_4_ association analyses. Blood samples of these cattle were collected near the end of the GEM system tests and a high performance four-channel chemical isotope labeling (CIL) liquid chromatography (LC) mass spectrometer (MS) method was applied to identify and quantify concentrations of metabolites. The four-channel CIL LC-MS method detected 4235 metabolites, of which 1105 were found to be significantly associated with AVG_DAILYCH4 by a t-test, while 1305 were significantly associated with AVG_DAILYCH4 by a regression analysis at p<0.05. Both the results of the t-test and regression analysis revealed that metabolites that were associated with enteric methane emissions in beef cattle were largely breed-specific whereas 4.29% to 6.39% CH_4_ associated metabolites were common across the three breed populations and 11.07% to 19.08% were common between two breed populations. Pathway analyses of the CH_4_ associated metabolites identified top enriched molecular processes for each breed population, including arginine and proline metabolism, arginine biosynthesis, butanoate metabolism, and glutathione metabolism for Angus; beta-alanine metabolism, pyruvate metabolism, glycolysis / gluconeogenesis, and citrate cycle (TCA cycle) for Charolais; phenylalanine, tyrosine and tryptophan biosynthesis, phenylalanine metabolism, arginine biosynthesis, and arginine and proline metabolism for Kinsella Composite. The detected CH_4_ associated metabolites and enriched molecular processes will help understand biological mechanisms of enteric methane emissions in beef cattle. The detected CH_4_ associated plasma metabolites will also provide valuable resources to further characterize the metabolites and verify their utility as biomarkers for selection of cattle with reduced methane emissions.

## Introduction

Climate change and global warming have been a concern for all humans since 1970s, and the main cause, greenhouse gas emissions, has attracted enormous attention with discussions in political, environmental, technological, and cultural areas [1]. Greenhouse gas mainly includes carbon dioxide (CO_2_), enteric methane (CH_4_), nitrous oxide (N_2_O), etc., in which the impact of CH_4_ on the climate change is more than 25 times greater than CO_2_. According to a widely quoted figure from United Nations Food and Agriculture Organization (FAO), livestock, particularly cattle, are responsible for 14.5 percent of global human-induced greenhouse gas emissions [2]. And due to an expected doubling demand of global milk and meat by 2050, CH_4_ emissions from livestock is predicted to substantially increase [3]. In Canada, it is estimated that beef cattle produce up to 20 million tons carbon dioxide equivalent (CO_2_ eq) of methane per year as a result of enteric fermentation, accounting for the great influence on the greenhouse effect [4].

Cattle release 6% of their ingested energy as eructated methane [5], thus reducing methane emissions not only can be beneficial in reducing carbon foot print of the industry, but also improve nutrition utilizing efficiency and decrease production costs. The mechanism of methane emissions from cattle remains to be fully revealed, but it has been shown that cattle methane emission is in part controlled by animal genetics, offering an opportunity to reduce methane emissions through genetic selection. Conventional genetic selection of low methane emission beef cattle based on directly measured phenotypes is challenging and expensive because methane emission data collection is time- and labor-intensive [6]. Marker assisted selection (MAS) or genomic selection is an alternative approach with identified predictive markers from DNA, transcriptomics, metabolomics or other types [7].

Of those, metabolomics is an emerging field and a powerful tool to characterize complex biochemical phenotypes. It has the potential to reveal promising biomarker candidates associated with biomolecular processes of methane emissions. The major issues of current metabolomics studies include low metabolome coverage as well as not high quantification accuracy. As a result, new techniques are required. Previously, we reported the use of high-performance chemical isotope labeling (CIL) LC-MS method for the profiling of amine/phenol submetabolome [8]. This derivatization method can significantly improve LC separation, MS sensitivity, and also provide precise relative quantification result. The power and promising future of this method have been realized from its successful application in various samples and areas [9–13]. In order to further improve metabolome coverage, recently, we have developed other CIL approaches targeting different submetabolomes, such as carboxylic acids [14], hydroxyls [15], ketones and aldehydes [16]. By using this divide-and-conquer strategy and combining all these four-channel methods together, we could gain a coverage as high as 90% of potential whole metabolome [17].

Thus, in this work, we applied the high performance four-channel CIL LC-MS method to detect and quantify plasma metabolites from cattle that were measured for enteric methane emissions. Subsequently, we conducted association analyses to identify metabolites and enriched molecular processes that are significantly associated with enteric methane emissions in beef cattle.

## Materials and methods

### Animal populations and management

Animals used in this study were from three beef cattle populations including Angus, Charolais, and Kinsella Composite (KC) that are located at the Roy Berg Kinsella Ranch, University of Alberta. The three beef cattle populations were previously described [18, 19]. Briefly, the Angus and Charolais cow herds were maintained via breeding using registered bulls with their pedigree information maintained by the Canadian Angus or Charolais association, respectively. The KC cow herd were produced from crosses among 3 composite cattle lines, namely beef synthetic 1, beef synthetic 2, and dairy × beef synthetic (DBS). The beef synthetic 1 was composed of 33% Angus, 33% Charolais, and about 20% Galloway, with the remainder from other beef breeds. The beef synthetic 2 composite was made up of about 60% Hereford and 40% other beef breeds. The dairy × beef synthetic was composed of approximately 60% dairy breeds (Holstein, Brown Swiss, or Simmental) and 40% beef breeds, mainly Angus and Charolais. The KC cow herd was maintained via breeding using Angus, Charolais, or University of Alberta Hybrid bulls that were produced from the three beef synthetic lines. All the three cow herds were bred between June to August and their calves were born between March to May. Calves remained with their dams until they were weaned at 6–7 months of age. All animals were raised and managed following the Canadian Council of Animal Care (CCAC) guidelines on the care and use of farm animals in research teaching and testing [20], and all the experimental procedures applied to the animals were approved by the University of Alberta Animal Care and Use Committee (AUP00000777).

### Methane emissions data collection

Methane emissions were measured on Angus and Charolais heifers after weaning and on KC mature cows using the GEM (GreenFeed emissions monitoring) system at the Roy Berg Kinsella Ranch along with a feedlot test trial. All the animals in the three breed population were fed ad libitum once daily (8am) with the mixed ration (as fed basis) during the feedlot test and during adaptation to diet (25 d before test) period. Ingredient composition of the mixed ration offered to animals in the Angus and Charolais populations was 71.8% silage, 19% oat grain, 4.5% corn dried distillers grains with solubles (cDDGS), and 4.7% Feedlot 30-Rumensin (F30). For the KC population, the ingredient composition was 85% Barley Silage, 10% Barley, 5% F30. The F30 contained crude Protein (min) 30.0%; Equivalent Crude Protein from Non-protein sources (max) 10.0%; Crude Fat (min) 2.5%; Crude Fibre (max) 8.0%; Flourine (max) 120 mg/kg; Sodium (act) 2,0%; Calcium (act) 8.5%; Phosphorus (act) 0.7%; Magnesium (act) 0.4%; Potassium (act) 0.9%; Sulfur (act) 0.4%; Iron (act) 135 mg/kg; iodine (act) 30 mg/kg; Copper (act) 310 mg/kg; Manganese (act) 840 mg/kg; Zine (act) 1,440 mg/kg; Cobalt (act) 6.0 mg/kg: Vitamin A (min) 100,000 LU./kg: Vitamin D (min) 10,000 I.U./kg: Vitamin E (min) 300 I.U./kg and monensin 22mg/kg.

Details of measuring methane emissions using the GEM system were described by Manafiazar et al. [21]. Briefly, the GEM system dispenses feed pellets to attract the animals. When an individual animal visits GEM, the system reads the animal’s radio-frequency identification tag, and the beginning, and end time of each visit are recorded. Any measurements with a minimum of 3 min per each visit time were considered as acceptable observations for monitoring emissions. Animals had free access to the GEM system 24 h/day while on drylot, although pellet intake was restricted such that each animal received a maximum of six drops per GEM visit, and pellets were dropped at 36 second intervals. After the sixth drop, animals were required to wait 4 h until their next six drops, resulting in a maximum of 36 drops per animal per day with six possible visits, and drop sizes averaged 35.4 g drop^−1^ (SD = 0.30). Pellets used in the GEM unit consisted of barley, beef vitamin-trace mineral premix, calcium carbonate, corn distiller’s grains screenings, sodium chloride, wheat/wheat middlings, and zinc chelate (MasterFeeds Inc., Red Deer, AB, Canada). Continuous and negative air flow from a system fan draw air past the animal’s nose and mouth when it enters the shroud, thus mixing air with respired and (or) eructated CH_4_ and CO_2_. This mixture was drawn up a collection pipe, remixed, sampled, and analyzed by a nondispersive infrared analyzer. Valid time per visit and visit duration were defined as any spot measurement of CH_4_ and CO_2_ emissions, where the animal’s head was continuously in the shroud within 20 cm of the proximity sensor for a minimum of 3 min. Total number of drops per day for each animal were also extracted. Visit data were then converted to daily emission data using SAS software program (SAS 2016) and measurements of individual animal enteric methane emissions were obtained as an average of daily CH_4_ amount in gram over the test period (i.e. AVG_DAILYCH4), which was expressed as (g d^−1^).

### Plasma sample collection and metabolite analyses

Near the end of GEM system tests, blood samples were collected in green topped lithium heparin vacutainers from each animal using jugular blood sampling once before morning feeding. There were no duplicates in the process of blood sample collection. Then, blood was thoroughly mixed with lithium heparin vacutainers and centrifuged to collect the plasma, which was then immediately frozen on dry ice, transported back to the lab and stored at -80°C for subsequent metabolite analyses. For this study, blood samples of 19 heifers born in 2017 from the Angus breed, 20 heifers born in 2017 from the Charolais breed, and 20 mature cows born in 2015 from the Kinsella Composite (KC) breed were used. These animals were selected from 72, 48, and 40 cattle measured for the enteric methane emissions in 2018 to represent cattle with the lowest or highest amounts of AVG_DAILYCH4 of each breed population.

### Chemicals and reagents

All the chemicals and reagents, unless otherwise stated, for metabolite analyses were purchased from Sigma-Aldrich Canada (Markham, ON, Canada). Dansyl chloride (DnsCl), p-dimethylaminophenacyl (DmPA) bromide, dansylhydrazine (DnsHz) and RT-calibrants was purchased from Nova Medical Testing (NovaMT) Inc (Edmonton, Alberta, Canada). LC−MS grade water, acetonitrile (ACN), and methanol (MeOH) were purchased from Thermo Fisher Scientific (Edmonton, Alberta, Canada).

### Analytical workflow

**Fig 1:**
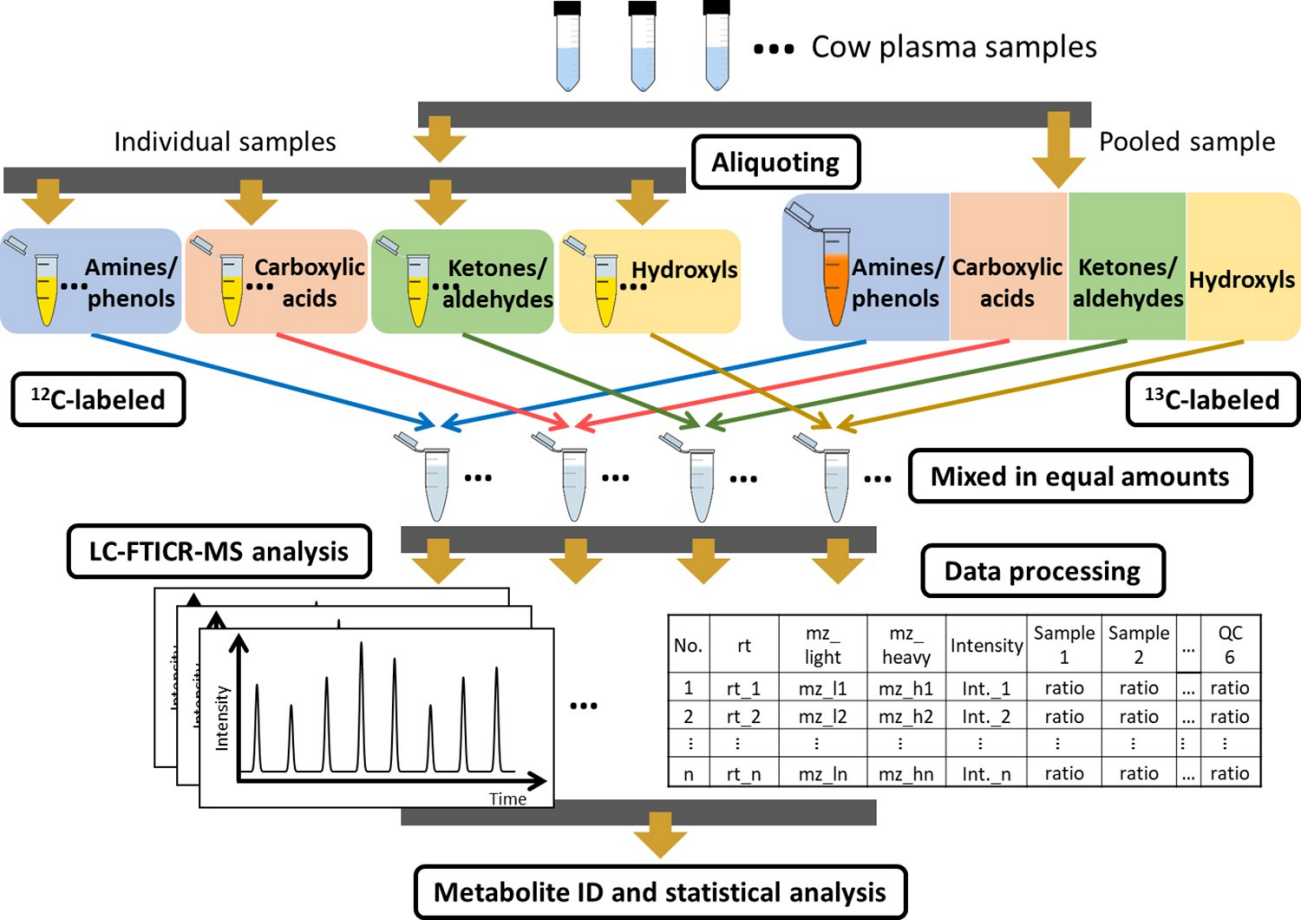
Workflow of 4-channel chemical isotope labeling LC-MS of cow plasma samples.

A high-performance chemical isotope labeling (CIL) method was applied to achieve relative metabolite quantification and high metabolome coverage. Fig 1 shows the overall workflow of this work. It contains the following steps: (1) sample aliquoting and generation of pooled sample, (2) 4-channel submebolome labeling, (3) LC-UV quantification and normalization, (4) mixing of ^12^C-labeled individual samples and ^13^C-labeled pooled sample at equal amounts, (5) LC-FTICR-MS analysis of ^12^C-/^13^C- mixtures, (6) data processing using R programs, (7) metabolite identification and statistical analysis using IsoMS Pro software (NovaMT, AB, Canada). The detailed experimental conditions are described below.

### Aliquoting and generation of the pooled sample

Each individual cow plasma sample from -80°C freezer was thawed then centrifuged at 15,000 g for 10 min. Supernatant was taken and split into 6 aliquots for four different labeling methods (15 μL each), backup (15 μL each) and preparation of pooled sample (35 μL each). For the one aliquot for preparing of pooled sample, all the aliquots from each individual sample were combined and mixed thoroughly, which was used as the reference. All of the leftovers were stored at -80°C for future analyses.

### Amine/Phenol submetabolome labeling

For the 15 μL aliquot for amine/phenol labeling, 45 μL of LC-MS grade MeOH was added to perform protein precipitation. The sample was vortexed then incubated at -20°C for 2 hours. After that, the sample was centrifuged at 15,000 g for 10 min. 45 μL of the supernatant was taken and completely dried using a Savant SC110A Speed Vac at room temperature. The sample was re-suspended in 25 μL of LC-MS grade water and ready for dansylation labeling. The labeling protocol was adapted from a previous report [8]. 25 μL of individual sample or 25 μL of the pooled sample was mixed with 12.5 μL of ACN. Then, 12.5 μL of 250 mM sodium carbonate/sodium bicarbonate buffer was added to the samples. The solution was mixed with 25 μL of freshly prepared ^12^C- dansyl chloride (DnsCl) solution (18 mg/mL, in ACN) (for light labeling, individual samples) or ^13^C-DnsCl solution (18 mg/mL, in ACN) (for heavy labeling, pooled sample). After incubation for 45 min at 40°C, 5 μL of 250 mM sodium hydroxide solution was added to quench the reaction. The solution was then incubated at 40°C for another 10 min. Finally, 25 μL of formic acid (425 mM) in ACN/water (50:50, v/v) was added to make the solution acidic.

### Carboxyl submetabolome labeling

For the 15 μL aliquot for carboxyl labeling, 45 μL of LC-MS grade ACN was added to perform protein precipitation. The sample was vortexed then incubated at -20°C for 2 hours. After that, the sample was centrifuged at 15,000 g for 10 min. 40 μL of the supernatant was taken and ready for DmPA labeling. The labeling protocol was adapted from a previous report [14]. 40 μL of individual sample or 40 μL of the pooled sample was mixed with 10 μL of 0.5 M triethanolamine. Then, 25 μL of freshly prepared ^12^C-DmPA solution (10 mg/mL, in ACN) (for light labeling, individual samples) or ^13^C-DmPA solution (10 mg/mL, in ACN) (for heavy labeling, pooled sample). After incubation for 60 min at 80°C, 20 μL of 0.2 M Tri-Gly was added and incubated at 80°C for another 30 min to quench the excessive labeling reagent.

### Hydroxyl submetabolome labeling

For the 15 μL aliquot for hydroxyl labeling, the labeling protocol was adapted from a previous report [15]. 3 μl of saturated NaCl and 1.5 μL of a 6 M HCl was added to the individual sample or the pooled sample. After vortexing, 45 μL of ethyl acetate was added to extract metabolite for twice. Then, the extracted solutions were combined and completely dried. After that, the sample was re-suspended in 12.5 μL of LC-MS grade ACN. 12.5 μl of freshly prepared 18 mg/mL ^12^C-(for light labeling, individual samples) or ^13^C- (for heavy labeling, pooled sample) DnsCl solution (in ACN) and 12.5 μL of 24.5 mg/mL DMAP solution (in ACN) was added. The sample was vortexed, followed by spinning down, and then incubated at 60°C for 60 minutes. After that, 2.5 μL of a 250 mM NaOH solution was added to quench the excessive labeling reagent. The solution was incubated at 60°C for another 10 minutes. Finally, 12.5 μL of 425 mM formic acid solution in ACN/water (50:50, v/v) was added to adjust pH.

### Carbonyl submetabolome labeling

For the 15 μL aliquot for carbonyl labeling, the labeling protocol was adapted from a previous report [16]. 45 μL of LC-MS grade MeOH was added to perform protein precipitation. The sample was vortexed, spun down, then incubated at -20°C for 2 hours. After that, the sample was centrifuged at 15,000 g for 10 min. 45 μL of the supernatant was taken and completely dried. The sample was re-suspended in 15 μL of LC-MS grade water. 15 μL of 144 mM HCl solution (in MeOH) was added to the individual sample or the pooled sample. After vortexing and spinning down, 15 μL of freshly prepared 20 mM ^12^C- (for the individual samples and the pooled sample) or ^13^C- (for the pooled sample) DnsHz (in MeOH) was added. The sample was then vortexed, followed by spinning down. The mixture was incubated at 40°C for 60 minutes. After that, the sample was removed from the incubator and placed in the -80°C freezer for 10 minutes to stop the reaction. The solution was then completely dried down. Finally, the labeled metabolites were re-dissolved in 80 μL of ACN/water (50:50, v/v).

### LC-UV sample normalization

The total amount of dansyl-labeled metabolites in each sample from amine/phenol labeling was measured using an LC-UV with a protocol reported previously [22]. The instrument used for detection was a Waters ACQUITY UPLC system with a photodiode array (PDA) detector. A Phenomenex Kinetex reversed-phase C18 column (50 mm × 2.1 mm, 1.7 μm, 100 Å pore size) was used to achieve a fast step-gradient. Mobile phase A was 0.1% (v/v) formic acid in 5% (v/v) ACN/water, and mobile phase B was 0.1% (v/v) formic acid in ACN. The gradient profile was as follows: t = 1 min, B = 0%; t = 1.01 min, B = 95%; t = 2.5 min, B = 95%; t = 3 min, B = 0%; t = 6 min, B = 0%. The flow rate was 0.45 mL/min. The peak area, which represents the total concentration of dansyl-labeled metabolites, was integrated using the Empower software (6.00.2154.003).

### Sample mixing

According to the quantification results, ^12^C-labeled individual samples and the ^13^C-labeled pooled sample were mixed in equal amounts, respectively, for all the 4 channels. ^12^C- and ^13^C-labeled pooled sample were mixed in equal amounts to serve as a quality control (QC) sample. Besides, for carboxyl, carbonyl and hydroxyl labeling, ^12^C- and ^13^C-labeled blanks (water) were mixed in equal volume to serve as the method blanks. The mixtures were ready for LC-MS analysis.

### LC-MS analysis

Labeled and mixed cow plasma samples were analyzed using a Bruker 9.4 T Apex-Qe FTICR mass spectrometer (Bruker, Billerica, MA), coupled with an Agilent capillary 1100 binary system (Agilent, Palo Alto, CA). An Agilent eclipse plus reversed-phase C18 column (100 × 2.1 mm, 1.8 μm) was used. Mobile phase A was 0.1% (v/v) formic acid in 5% (v/v) ACN/water, and mobile phase B was 0.1% (v/v) formic acid in ACN. The gradient profile for amine/phenol labeling was: t = 0 min, 20% B; t = 3.5 min, 35% B; t = 18 min, 65% B; t = 24 min, 99% B; t = 32 min, 98% B. The gradient profile for carboxyl labeling was: t = 0 min, 20% B; t = 9 min, 50% B; t = 22 min, 65% B; t = 26 min, 80% B; t = 28 min, 98% B; t = 34 min, 98% B. The gradient profile for hydroxyl labeling was: t = 0 min, 20% B; t = 3.5 min, 35% B; t = 9.2 min, 65% B; t = 21.2 min, 99% B; t = 31.2 min, 99% B. The gradient profile for carbonyl labeling was: t = 0 min, 1% B; t = 3min, 25% B; t = 23min, 99% B; t = 34min, 99% B. Flow rate was all 180 μL/min. All mass spectra were collected in the positive ion mode. The MS conditions for FTICR-MS were: nitrogen nebulizer gas, 2.3 L/min; dry gas flow, 7.0 L/min; dry temperature, 195°C; capillary voltage, 4200 V; spray shield, 3700 V; acquisition size, 256 k; mass scan range, m/z 200−1000; ion accumulation time, 1 s; TOF (AQS), 0.007 s; DC extract bias, 0.7 V. All the samples were injected in random order. QC samples, blanks and RT-calibrants were injected every 10 runs to monitor the performance of the instrument.

### Data processing

After LC-FTICR-MS analysis, the entire list of centroid peaks with information of retention times, m/z values and peak intensities was exported to.csv files using Bruker Data Analysis software (Version 4.0). IsoMS [23] R-program was used to pick peak pairs, filter false-positive pairs (e.g., dimers and common adducts) and calculate peak-pair relative intensity ratios. After the alignment of peak pairs from multiple samples using the Alignment R-program, the Zerofill R-program was applied to recover the high-confidence peak pair ratios lost during the previous data processing steps. For carboxyl, carbonyl and hydroxyl labeling, Blank Subtraction R-program was used to reduce background peak pairs.

### Metabolite identification

Three-tiers identification approach was used to perform metabolite identification using IsoMS Pro software. In tier 1, based on accurate mass and retention time, peak pairs were searched against a labeled metabolite library-CIL Library, which contains more than 1,300 experimental entries, including metabolites and dipeptides. In tier 2, based on accurate mass and predicted retention time, the remaining peak pairs were searched against a linked identity library (LI Library), which includes over 7,000 pathway-related metabolites and provides high-confidence putative identification results. In tier 3, based on accurate mass, the remaining peak pairs were searched against the MyCompoundID (MCID) library, which is composed of 8,021 known human endogenous metabolites (zero-reaction library) and their predicted metabolic products from one metabolic reaction (375,809 compounds) (one-reaction library) and two metabolic reactions (10,583,901 compounds) (two-reaction library).

### Methane emission and metabolite data consolidation

AVG_DAILYCH4 and each metabolite concentration were examined for outliers and normality of distribution. Values that are greater or smaller than 3 times standard deviation of the mean of AVG_DAILYCH4 or metabolite concentration were excluded. Descriptive statistics of original phenotypic values of AVG_DAILYCH4 and all metabolites were provided in S1 and S2 Tables, respectively. Since animal ages at the start of measuring CH_4_ and thus at blood sample collection were different, the values of AVG_DAILYCH4 and all metabolite concentrations were adjusted through a regression on the animal age factor using glm function in R programming. The regression model is shown below.


$$y=\beta_{0}+\beta_{1}*X_{1}+\varepsilon$$ (1)

Where *y* is the vector of the original phenotype values, *β*_0_ is the intercept, *β*_1_ is the slope of the line, *X*_1_ is the animal age in day, *ε* is the residual term. The residuals of the regression were used as adjusted phenotypic values for further analyses. The adjusted CH_4_ (AVG_DAILYCH4) and adjusted metabolite concentrations conform to a normal distribution for each breed population as shown in normal quantile–quantile (Q-Q) plots of S1 Fig (adjusted AVG_DAILYCH4 and adjusted metabolite A-5 as an example).

### Association analyses

**T-test within breed population.** The animals were assigned into high or low methane emission groups within each breed based on their adjusted AVG_DAILYCH4 values. The high and low methane emissions groups had significantly different AVG_DAILYCH4 values in each of the Angus (p-value = 4.51E-05), Charolais (p-value = 2.81E-04), and KC (p-value = 3.67E-09) breed populations as shown in Table 1.

**Table 1:** Significance of differences of AVG_DAILYCH4 (g/per day) between the high and low methane emission groups in the Angus, Charolais, and Kinsella Composite (KC) populations.

Threshold	Number of records	Numb er of records in low group	Number of records in high group	Mean_low group±se	Mean_high group±se	p-value
**Angus**	19	10	9	-16.5±2.97	18.33±5.03	4.51E-05
**Charolais**	20	10	10	-13.59±4.92	13.59±1.62	2.81E-04
**KC**	20	10	10	-31.08±4.2	31.08±4.1	3.67E-09

A t-test of adjusted metabolite concentrations between the high and low methane emission groups within each breed was conducted to detect metabolites that are significantly associated with the amount of methane emission. In order to consider multiple testing, permutation tests were used to determine the significance threshold value (p-value) for the t-test [24]. In the permutation tests, adjusted metabolite concentrations were randomly shuffled for each metabolite and then assigned to animals in the high or low methane groups, and a t-test was conducted and the corresponding p-value was recorded. The process was repeated 1000 times and 1000 p-values were recorded. Then, all the recorded 1000 p-values were ordered from smallest to largest and the 50th p-value, which corresponds to the 5% type 1 error of the order, was used as the significance threshold value of the t-test for each corresponding metabolite.

#### Construction of volcano plots within breed population

In order to integrate both the biological and statistical significance concerning the metabolites, volcano plots were implemented within each breed population. In statistics, a volcano plot is a type of scatter-plot that is used to quickly identify changes in large data sets composed of replicate data [25, 26]. In our study, volcano plots combined a measure of statistical significance (p-value) from the t-test above (between the high and low methane emission groups within each breed) with the magnitude of the change, enabling quick visual identification of those data-points (metabolites, etc.) that display large magnitude changes that are also statistically significant. The fold change (FC) of volcano plots were calculated as Mean(High group) / Mean(Low group), and the metabolites selection criteria of FC was 1 <|*Log*_2_*FC*| 6.644 (2^6.644^≈100). Different from general volcano plots, we used the significance threshold value obtained from permutation tests to determine the significance of t-test in our volcano plots. The volcano plots was conducted using Metaboanalyst 5.0 and R program.

#### Regression analyses within breed population

A regression analysis of adjusted AVG_DAILYCH4 to each adjusted metabolite concentration was also performed to detect metabolites that are significantly associated with the amount of methane emission. Similarly, permutation tests were used to determine the significance threshold value (p-value) at the 5% type error for the regression analysis [24].

### Pathway analyses

Metabolites that were detected by both the t-test and the regression analysis as having significant associations with the enteric methane emissions in each breed population were listed for subsequent pathway analysis. Pathway analysis was conducted using Metaboanalyst 5.0 for those common detected metabolites that have a high-confidence of quantification (tier 1 and tier 2). Those commonly detected enteric methane emission associated metabolites were matched to the *Bos taurus* (cow) (KEGG) pathway library to identify enriched molecular processes.

## Results

### Metabolite detection and identification results of four-channel labeling LC-MS

The four-channel labeling technique targets amine/phenol -, carboxyl-, hydroxyl-, and carbonyl—submetabolomes. The data was firstly processed for each submetabolome separately, and then combined together. A total of 4235 peak pairs or metabolites were detected for the 59 cow plasma samples. Three-tier identification approach was used for identifying these metabolites. In tier 1, a total of 110 peak pairs were positively identified against CIL library. In tier 2, 260 peak pairs were putatively identified with high confidence against LI library. In tier 3, 676, 1709 and 941 peak pairs were matched in the zero-, one- and two-reaction libraries, respectively, against MCID library. Thus, in total, out of 4235 detected metabolites, 3696 metabolites (87.3%) were either definitely or putatively identified. Among them, 370 metabolites were identified as high-confidence results (tier 1 and tier 2), which were more important as they can be used for other analysis (e.g., pathway analysis). The detailed metabolite detection and identification results were presented in S3 Table.

### Metabolites associated with enteric methane emissions detected by the t-test and volcano plots in the three populations

**Fig 2:**
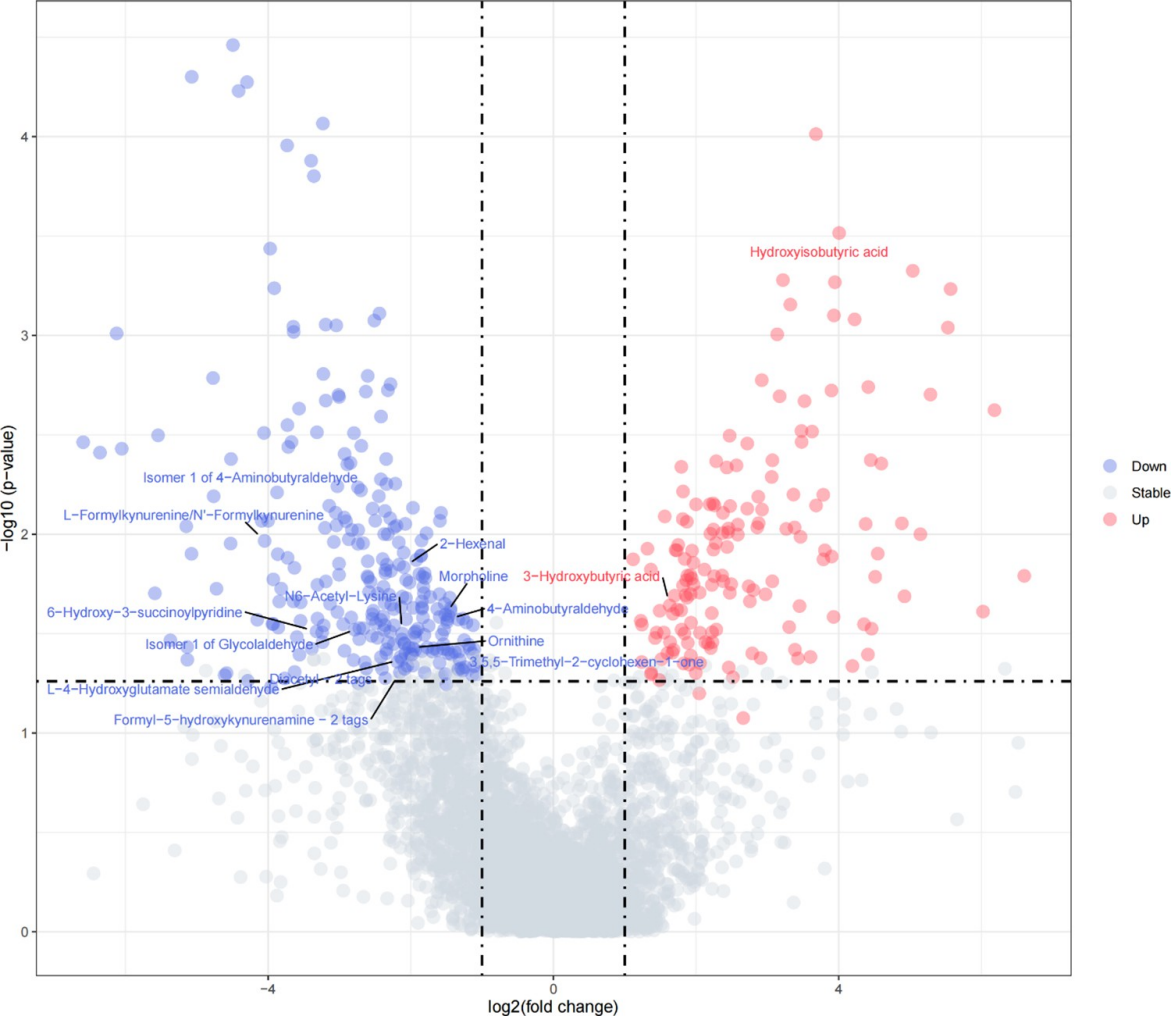
The volcano plot of differential metabolite identification between the high and low enteric methane emission (AVG_DAILYCH4) groups in the Angus population.

**Fig 3:**
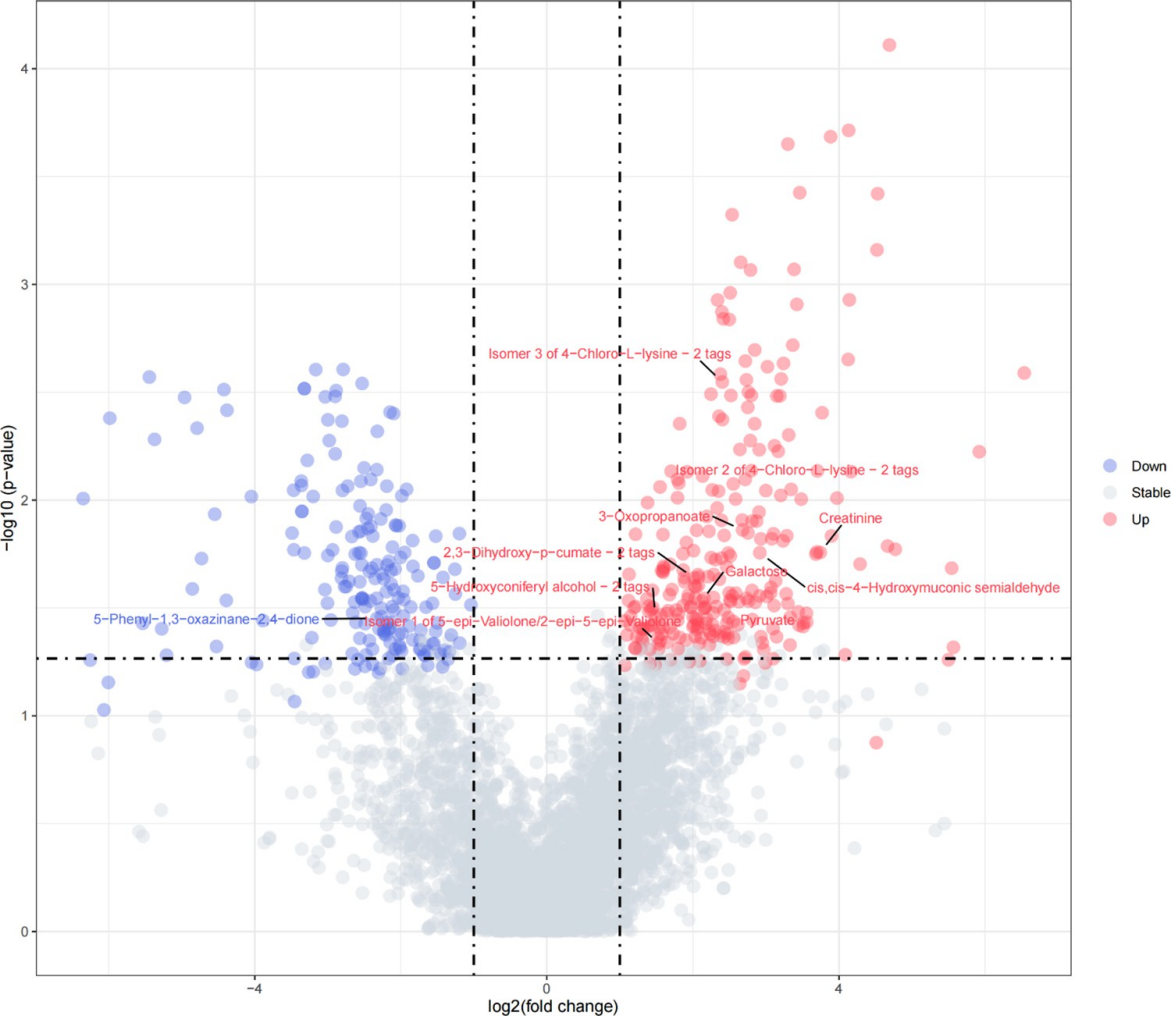
The volcano plot of differential metabolite identification between the high and low enteric methane emission (AVG_DAILYCH4) groups in the Charolais population.

**Fig 4:**
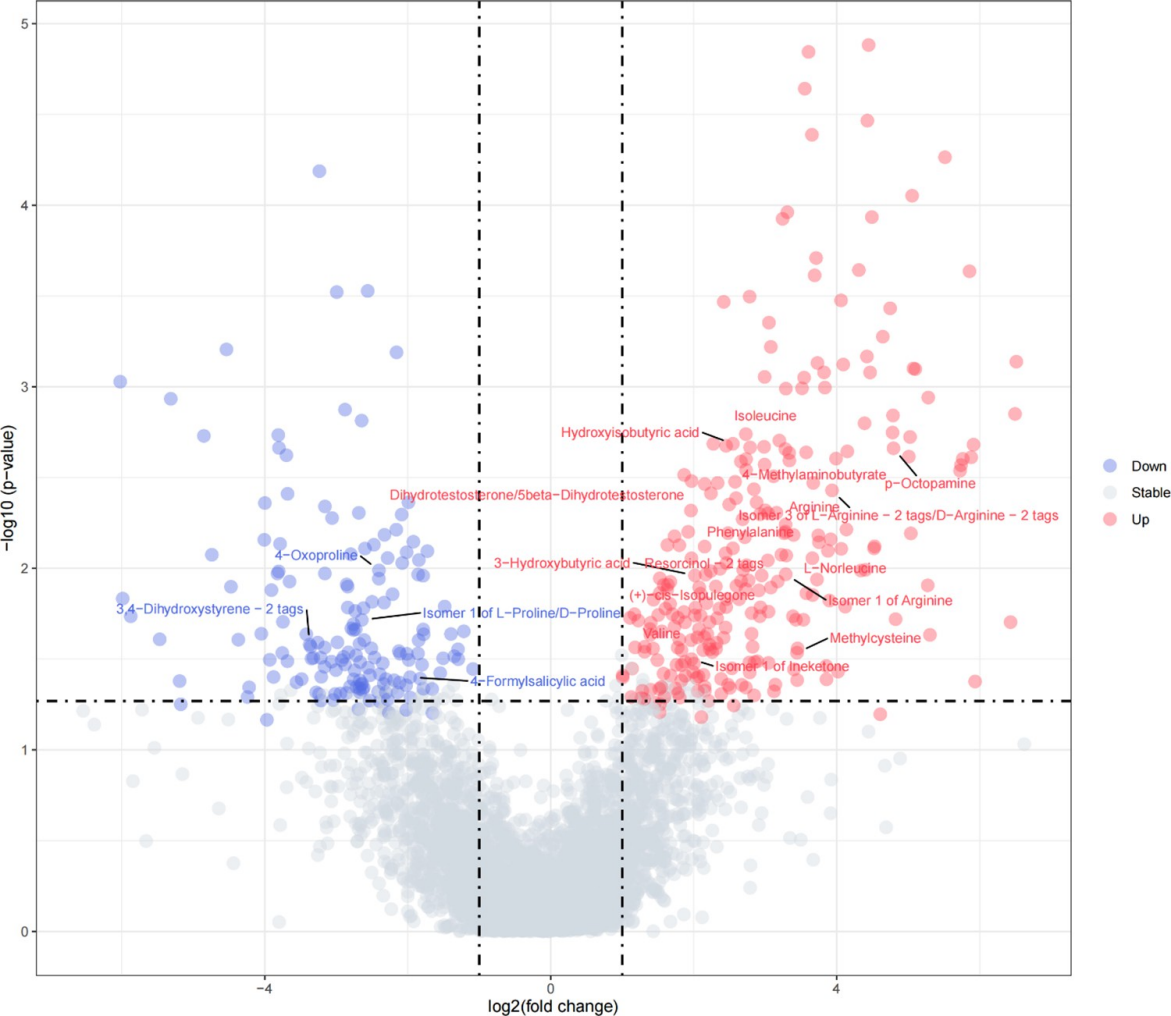
The volcano plot of differential metabolite identification between the high and low enteric methane emission (AVG_DAILYCH4) groups in the Kinsella Composite (KC) population.

Table 2 showed the number of metabolites that were significantly associated with CH_4_ detected by the t-test and a complete list of metabolites names was presented in S4 Table. As summarized in Table 2, there were 1406 significant metabolites, of which 1105 were unique, from the three populations using the t-test method, with 458, 476 and 472 metabolites for Angus, Charolais and KC, respectively. And the p-value ranges were from 3.46E-05 to 9.75E-02, 7.76E-05 to 1.33E-01 and 6.12E-09 to 6.83E-02 for Angus, Charolais and KC, respectively. The volcano plots of differential metabolite identification between the high and low enteric methane emission (AVG_DAILYCH4) groups in the Angus, Charolais and KC populations were shown in Figs 2–4. As is shown in Table 2, the Log_2_FC range used for volcano plots were from -6.5918 to -1.0448 and 1.1173 to 6.6009, -6.3489 to -1.0396 and 1.0685 to 6.5368, -6.0214 to -1.0871 and 1.0063 to 6.5121 for Angus, Charolais and KC, respectively. Additional, S5 Table presented a list of the Log_2_FC, pvalues and directions for enteric methane emissions of each metabolite for the volcano plots in the Angus, Charolais, and Kinsella Composite (KC) populations. For Angus, Charolais and KC, 157, 266 and 262 differential metabolites were positively correlated with the CH_4_ amount, and 265, 195 and 157 differential metabolites were negatively correlated with the CH_4_ amount. Also, the number of metabolites that are significantly associated with the enteric methane emissions (AVG_DAILYCH4) detected by volcano plots were shown in Table 2, it is noticed that the volcano plots verified 422 of 458 (92.1%), 461 of 476 (96.8%) and 419 of 472 (88.8%) of the metabolites detected by the t-test in the three breed populations, which means the t-test detected additional 36, 14, and 53 metabolites in the three populations, respectively. All the metabolites identified in volcano plots were included in t-test results. As the vast majority of the metabolites detected by the t-test in the three breed populations (88.8% to 96.8%) were verified through the volcano plots and the t-test identified additional metabolites associated with CH_4_, we will focus on all the potential CH_4_ associated metabolites detected by the t-test for subsequent analysis and discussion.

**Table 2:** Number of metabolites that are significantly associated with the enteric methane emissions (AVG_DAILYCH4) detected by the t-test and volcano plot in the Angus, Charolais, and Kinsella Composite (KC) populations.

Population	T-test	p-value range	Permutation p-value threshold range	Volcano plot	Log_2_FC range	Common between T-test and Volcano plot
**Angus**	458	3.46E-05- 9.75E-02	3.08E-02- 1.33E-01	422	(-6.5918~-1.0448) &(1.1173~6.6009)	422
**Charolais**	476	7.76E-05- 1.33E-01	3.84E-02- 1.75E-01	461	(-6.3489~-1.0396) &(1.0685~6.5368)	461
**KC**	472	6.12E-09- 6.83E-02	3.53E-02- 1.04E-01	419	(-6.0214~-1.0871) &(1.0063~6.5121)	419
**Total**	1406	-	-	1302	-	1302

**Fig 5:**
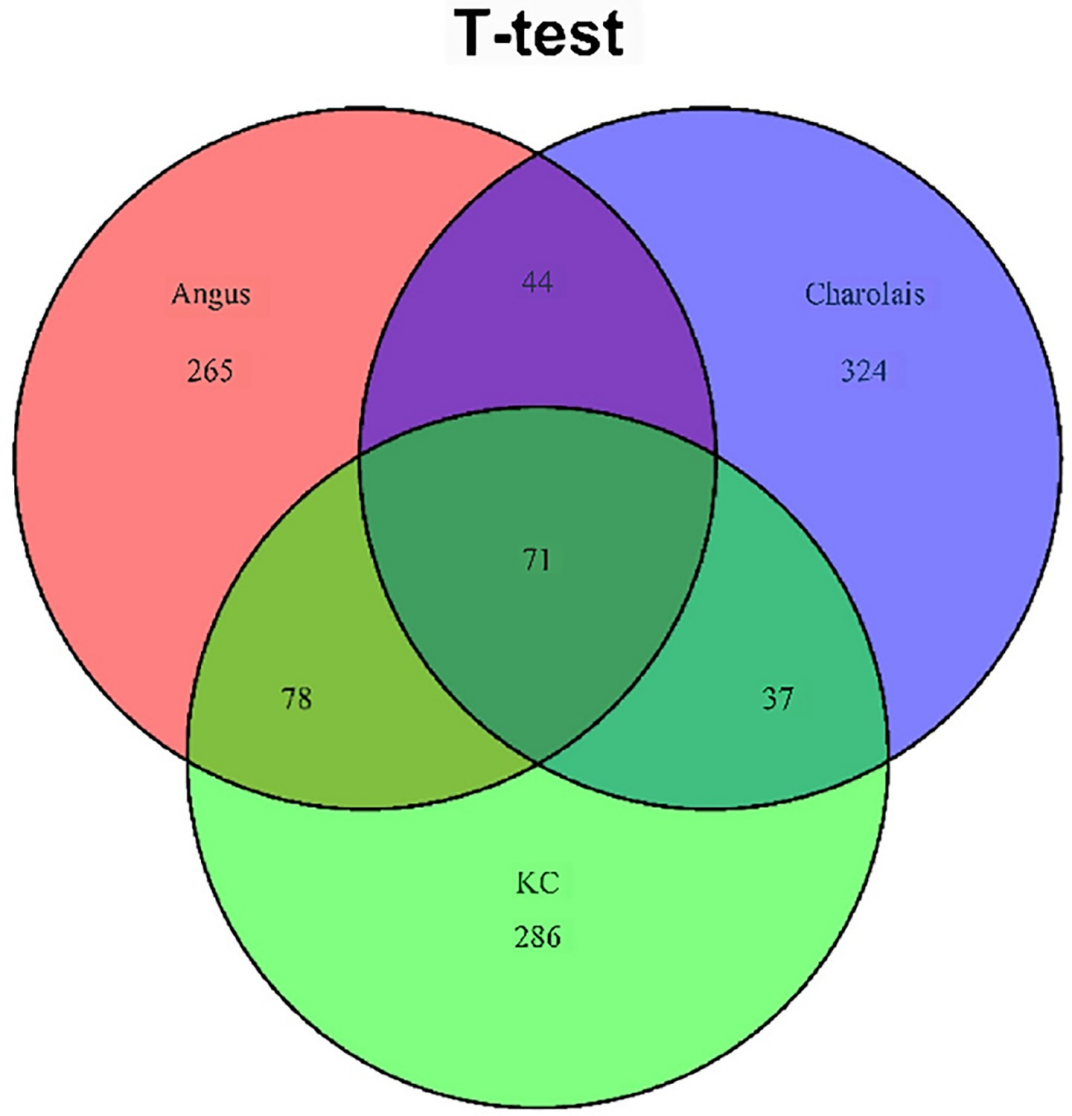
Venn diagram showing overlap of metabolites significantly associated the enteric methane emissions (AVG_DAILYCH4) at p<0.05 based on the t-test in the Angus, Charolais, and Kinsella Composite (KC) populations.

The number of common metabolites between different breed populations that were significantly associated with CH_4_ detected by the t-test was shown in Table 3, in which 115 (14.04%) significant metabolites were common between Angus and Charolais, 149 (19.08%) significant metabolites were common between Angus and KC, and 108 (12.86%) significant metabolites were common between Charolais and KC. A Venn diagram of significant metabolites from the results of the t-test in different breed populations was shown in Fig 5, of which, 71 metabolites (6.39%) were common among the three breed populations. As is shown in S4 Table, all the 71 common metabolites had the same direction of correlations for CH_4_ amount for the Angus and KC populations, of which 66 of the 71 metabolites were positively correlated with the CH_4_ amount, and 5 of the 71 metabolites had the negative correlations with the CH_4_ amount. Of the 71 common significant metabolites in Charolais, 66 had the negative correlations with the CH_4_ amount, and 5 metabolites had the positive correlations with the CH_4_ amount. However, the direction of correlations between the significant metabolites and CH_4_ amount in Charolais was opposite than that in Angus and KC.

**Table 3:** Number of common metabolites between or among the Angus, Charolais, and Kinsella Composite (KC) populations that are significantly associated with the enteric methane emissions (AVG_DAILYCH4) detected by both the t-test and regression analysis.

Combination	T-test	Regression
**Common between Angus and Charolais**	115	131
**Common between Angus and KC**	149	177
**Common between Charolais and KC**	108	94
**Common among three population**	71	56

### Metabolites associated with enteric methane emissions detected by regression analysis in the three populations

The number of metabolites that were significantly associated with CH_4_ detected by regression was shown in Table 4 and a complete list of metabolites names was also presented in S4 Table. In total, 1651 significant metabolites, of which 1305 were unique, were discovered using the regression analysis in the three populations with 708, 478, and 465 significant metabolites in Angus, Charolais and KC, respectively. The p value ranges in the three populations were from 1.68E-10 to 6.26E-02, 4.86E-06 to 6.49E-02 and 1.00E-08 to 5.79E-02, respectively.

**Table 4:** Number of metabolites that are significantly associated with the enteric methane emissions (AVG_DAILYCH4) detected by the regression analysis in the Angus, Charolais, and Kinsella Composite (KC) populations.

Population	Regression	p-value range	Permutation p-value threshold range
**Angus**	708	1.68E-10- 6.26E-02	3.08E-02- 8.70E-02
**Charolais**	478	4.86E-06- 6.49E-02	2.91E-02- 7.13E-02
**KC**	465	1.00E-08- 5.79E-02	3.25E-02- 7.34E-02
**Total**	1651	-	-

**Fig 6:**
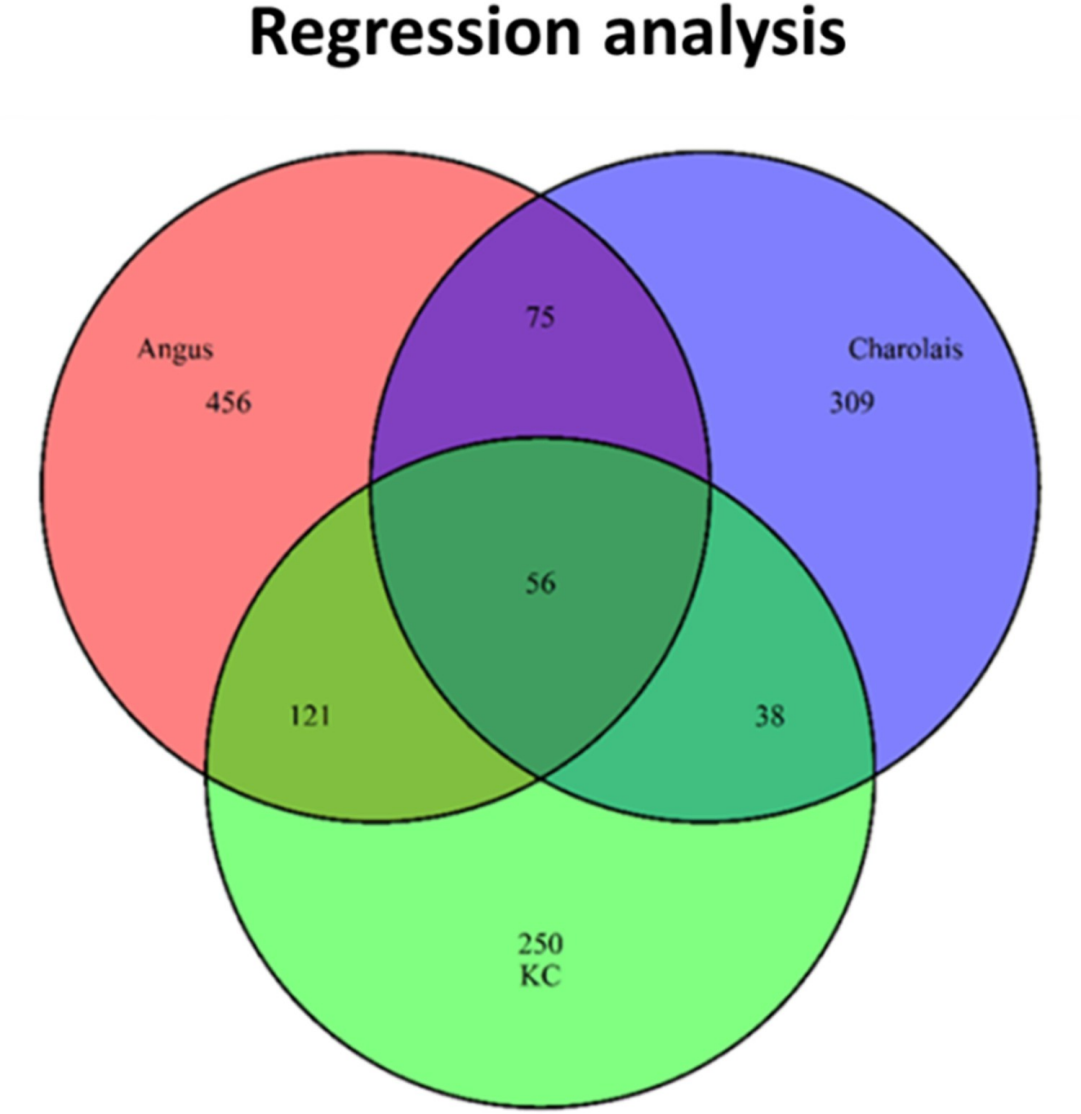
Venn diagram showing overlap of metabolites significantly associated the enteric methane emissions (AVG_DAILYCH4) at p<0.05 based on the regression analysis in the Angus, Charolais, and Kinsella Composite (KC) populations.

As is shown in Table 3, 131 (12.42%) significant metabolites were common between Angus and Charolais, 177 (17.77%) significant metabolites were common between Angus and KC, and 94 (11.07%) significant metabolites were common between Charolais and KC. The Venn diagram (Fig 6) showed that 56 (4.29%) of them were common metabolites across the three breed populations. As presented in S4 Table, for Angus and KC, 49 of the common 56 metabolites were positively correlated with the CH_4_ amount and 7 of the common 56 metabolites were negatively correlated with the CH_4_ amount, and they had the same direction of correlations. For Charolais, 50 of the 56 common metabolites were negatively correlated with the CH_4_ amount and 6 metabolites were positively correlated with the CH_4_ amount. Of these 56 common metabolites in Charolais, 55 metabolites had opposite correlation directions that that in Angus and KC.

The t-test method detected 265, 324, and 286 significant metabolites that were specific to the Angus, Charolais, and KC population, which represents approximately 57.9%, 68.1%, 60.6% of the significant metabolites detected in the breed population, respectively. The regression analyses discovered 456, 309, and 250 significant metabolites that were specific to the Angus, Charolais, and KC population, which represents approximately 64.4%, 64.6%, 53.8% of the significant metabolites detected in the breed population, respectively.

### Comparison between the results of t-testand regression analysis

**Fig 7:**
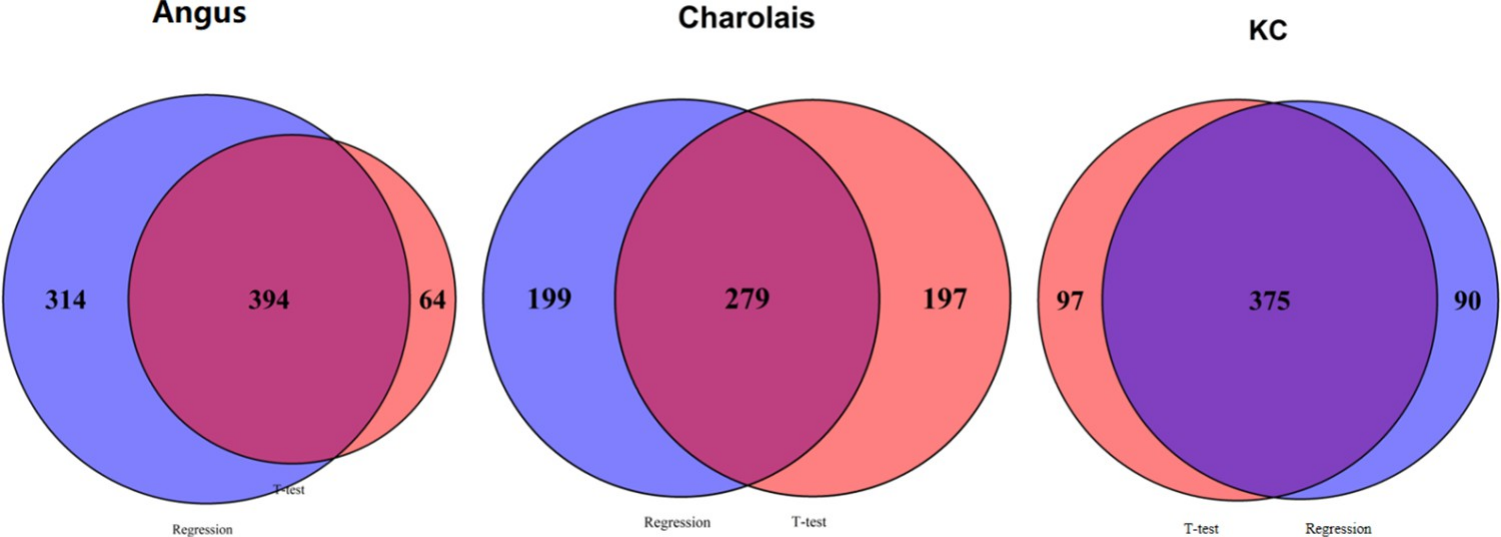
Venn diagram showing overlap of metabolites significantly associated the enteric methane emissions (AVG_DAILYCH4) between the t-test and regression analysis at p<0.05 in the Angus, Charolais, and Kinsella Composite (KC) populations.

We used both the t-test and regression to detect metabolites that are significantly associated with CH_4_. As is shown in Table 5 and Fig 7, there were 394 (51%), 279 (41.3%), and 375 (66.7%) common significant metabolites between the t-test and regression analysis in the Angus, Charolais and KC populations, respectively. It was found that 64 (8.3%), 197 (29.2%), and 97 (17.3%) metabolites were detected by t-test but not by regression in the Angus, Charolais and KC populations, respectively. A total of 314 (40.7%), 199 (29.5%), and 90 (16%) metabolites were detected by regression but not by t-test in Angus, Charolais and KC, respectively. We used a high performance four-channel CIL LC-MS method in this study and were able to quantify 4235 peak pairs or metabolites using a 3-tier identification approach. We further showed the significant metabolites of high-confidence metabolite quantification (tier 1 and tier 2) detected by both the regression analysis and t-test in the three cattle populations in Table 6. As shown in Table 6, 15, 12 and 21 high-confidence detected metabolites (tier 1 and tier 2) for Angus, Charolais, and KC populations, respectively, were significantly associated with methane emissions.

**Table 5:** Number of candidate metabolites associated with methane emissions (AVG_DAILYCH4) that were detected by both the t-test and regression analysis for the Angus, Charolais, and Kinsella Composite (KC) populations and suggestive metabolites that were detected by one of the t-test and regression methods.

Population	T-test	Regression	Common (Identified)	T-test_not_regression^a^	Regression_not_t-test^b^	Suggestive
**Angus**	458	708	394	64	314	378
**Charolais**	476	478	279	197	199	396
**KC**	472	465	375	97	90	187

^a^The number of metabolites identified by t-test but not identified by regression analysis.

^b^The number of metabolites identified by regression analysis but not identified by t-test.

**Table 6:** Common metabolites of Tier 1 and Tier 2 detected to be associated with the enteric methane emissions (AVG_DAILYCH4) in the Angus, Charolais, and Kinsella Composite (KC) populations using both the t-test and regression analysis.

Angus	Direction	Charolais	Direction	KC	Direction
N6-Acetyl-Lysine	-	Galactose	+	Isomer 1 of Arginine	+
Ornithine	-	2,3-Dihydroxy-p-cumate—2 tags	+	Arginine	+
**3-Hydroxybutyric acid**	+	5-Hydroxyconiferyl alcohol—2 tags	+	Methylcysteine	+
**Hydroxyisobutyric acid**	+	Creatinine	+	Valine	+
Formyl-5-hydroxykynurenamine—2 tags	-	Isomer 2 of 4-Chloro-L-lysine—2 tags	+	Phenylalanine	+
L-4-Hydroxyglutamate semialdehyde	-	Isomer 3 of 4-Chloro-L-lysine—2 tags	+	Isoleucine	+
Isomer 1 of 4-Aminobutyraldehyde	-	cis,cis-4-Hydroxymuconic semialdehyde	+	**3-Hydroxybutyric acid**	+
4-Aminobutyraldehyde	-	5-Phenyl-1,3-oxazinane-2,4-dione	-	**Hydroxyisobutyric acid**	+
**L-Formylkynurenine/N’-Formylkynurenine**	-	Isomer 1 of 5-epi-Valiolone/2-epi-5-epi-Valiolone	+	Resorcinol—2 tags	+
Morpholine	-	3-Oxopropanoate	+	3,4-Dihydroxystyrene—2 tags	-
2-Hexenal	-	Pyruvate	+	**L-Formylkynurenine/N’-Formylkynurenine**	+
Diacetyl—2 tags	-	Isomer 1 of 2-Oxobutanoate	+	Isomer 3 of L-Arginine—2 tags/D-Arginine—2 tags	+
3,5,5-Trimethyl-2-cyclohexen-1-one	-			Isomer 1 of L-Proline/D-Proline	-
Isomer 1 of Glycolaldehyde	-			4-Methylaminobutyrate	+
6-Hydroxy-3-succinoylpyridine	-			p-Octopamine	+
				L-Norleucine	+
				Dihydrotestosterone/5beta-Dihydrotestosterone	+
				4-Oxoproline	-
				Isomer 1 of Ineketone	+
				(+)-cis-Isopulegone	+
				4-Formylsalicylic acid	-

+: The metabolite is positively associated with amount of CH_4_

-: The metabolite is negatively associated with amount of CH_4_

### Pathway analysis

A list of enriched molecular process with p-values and impact factors from the pathway analyses was presented in S6 Table for each breed population. As an illustration, plots of -log (p-value) against the pathway impact of top four enriched molecular process were shown in S2A, S3A, and S4A Figs, respectively, for Angus, Charolais, and KC with the pathway of the highest impact and significance located on the top right corner. On the pathway schematic (S2B, S3B and S4B Figs), light blue means the metabolites cannot be identified, but it was used as a background for enrichment analysis. Those metabolites with other colors (varying from yellow to red) were the positively identified metabolites with different levels of significance. The red-colored metabolite had a more significant change between the low and high methane emission groups than a yellow-colored metabolite. The relative metabolite concentrations of those metabolites in the low and high methane emission groups were also displayed. The top four enriched molecular processes detected by the pathway analysis for each breed population included arginine and proline metabolism, arginine biosynthesis, butanoate metabolism, and glutathione metabolism for Angus (S2 Fig), beta-alanine metabolism, pyruvate metabolism, glycolysis / gluconeogenesis, and citrate cycle (TCA cycle) for Charolais (S3 Fig), phenylalanine, tyrosine and tryptophan biosynthesis, phenylalanine metabolism, arginine biosynthesis, and arginine and proline metabolism for KC (S4 Fig). Arginine and proline metabolism and arginine biosynthesis were two common pathways detected by the both Angus and KC populations.

## Discussion

The high performance four-channel CIL LC-MS method was able to detect and quantify up to 4235 metabolites in cattle plasma samples. This high level coverage of metabolites provides a great resource to investigate biological basis of complex traits in cattle. Indeed the analyses identified more than one thousands of metabolites in total that showed significant associations with the enteric methane emissions in the three beef breed populations. However, both the results of the t-test and regression analysis revealed that methane emission associated metabolites in beef cattle were largely breed-specific. The difference of metabolites across samples was further tested by a sparse partial least squares-discriminant analysis (sPLS-DA) [27, 28]that investigated distributions of samples with low CH_4_ emission measurements in regarding to their metabolite concentrations in the three beef cattle population. The sPLS-DA results (S5 Fig) showed a clear separation of the three breed populations, indicating that there were significant metabolomic differences across the three breed populations.

The results of breed-specific metabolite and methane emission associations were consistent with previous reports in RNAseq studies. Robert Mukiibi et.al investigated the hepatic transcriptomic profiles and their associations with ADG, DMI, RFI and MWT in the same Angus, Charolais, and Kinsella Composite (KC) populations through global RNAseq analyses and found that the identified differentially expressed (DE) genes were largely breed-specific [29, 30]. Similar results were found for rumen microbiota, and some studies have also indicated that the rumen microbiota could be influenced by host breed/species in ruminants [31, 32]. In addition, Zhang et.al assessed the effects of breed and feed efficiency on rumen microbiota and demonstrated that cattle breed could affect rumen microbiota at both the abundance and activity level [33].

The Angus and KC populations have more common metabolites that are significantly associated with CH_4_ compared with the Angus and Charolais or Charolais and KC populations although animals in the Angus and Charolais populations were all heifers and had the same diet/management levels whereas animals in the KC population were mature cows and were fed a diffident diet. It is noted that the Angus and Charolais are pure breeds while the KC population is a composite herd. Based on our breed composition analyses using DNA genotypes, 20 KC cows have 33.41% and 0.57% Angus and Charolais, respectively, plus other breeds. The greater proportion of Angus in the KC population indicates that the KC cattle are more genetically related to Angus than to Charolais, which supports the results that more significant metabolites were shared between the KC and Angus populations. Angus, a British breed, is characterized by its moderate frame and earlier fattening, which allows it to accumulate fat at an early stage, whereas Charolais is a continental European breed and is characterized by a larger frame and later maturity to fattening [34]. It is reported that in comparison with the Charolais breed, Angus has greater fat depth, greater marbling score and more daily DMI but less ADG at the similar ages [18, 35], presumably due to early maturity in Angus allowing the cattle to deposit more fat at a younger age [36] and in growing cattle, more energy is needed to deposit fat than protein because protein synthesis is energetically more efficient than fat synthesis [37, 38]. The distinctive biological processes between Angus and Charolais may explain why the directions of associations between the significant metabolites and methane emission detected in this study were largely different than that in the Angus and KC populations.

The discrepancies of the association analyses results between the t-test and the regression are likely due to the relatively small sample size used. Published reviews on the fundamental factors determining an appropriate sample size reported that sample size determination is contingent upon the significance threshold and the type of statistical test [39, 40]. In order to find the reason for the different results between the t-test and regression analysis in our study, the scatter plots for some uncommon significant metabolites concentration vs. CH_4_ amount were presented in S6 Fig. When the relationship between the adjusted AVG_DAILYCH4 (g/per day) and metabolite concentrations is sufficiently nonlinear, which may be also due to a small sample size, the regression analysis will likely not detect the metabolite at the significance level. The small sample size may also reduce the power of detecting significant metabolites by the t-test that contrasts the means of metabolite concentrations between the low and high methane groups, but the regression analyses may detect the significant association as it regresses metabolite concentrations on methane emission amounts on all animals.

However, the small sample size should have a smaller impact in detecting metabolite and methane emissions when its association is stronger. In this study, we further examined the specific directions of metabolites and CH_4_ correlations as in the S4 Table. The results suggested that the directions of all the 394, 279, and 375 common metabolite-CH_4_ associations detected by the t-test are the same as that detected by the regression in Angus, Charolais and KC, respectively. Therefore, in consideration of limitations of the smaller sample size used, we further classified metabolites that were detected by both the t-test and by regression as candidate or common metabolites associated with methane emission and metabolites that were detected by one of the t-test and regression methods as suggestive. We found 394, 279 and 375 candidate or common metabolites for methane emission in Angus, Charolais and KC, and 378, 396 and 187 suggestive metabolites associated with methane emission in Angus, Charolais, and KC, respectively.

Metabolites 3-Hydroxybutyric acid (C-1373), Hydroxyisobutyric acid (C-1580) and L-Formylkynurenine/N’-Formylkynurenine (A-357) were detected in both the Angus and KC populations in Table 6. And as shown in S4 Table, 3-Hydroxybutyric acid and Hydroxyisobutyric acid had positive correlations with the CH_4_ amount, and L-Formylkynurenine/N’-Formylkynurenine had a negative correlation with CH_4_ amount. Minji Kim et.al [41] investigated the metabolic characteristics of Japanese Black cattle using enteric methane emissions and found that in cattle with high methane emissions the concentration of blood β-hydroxybutyric acid and insulin levels were high, whereas blood amino acid levels were low, which is similar to our results. Luz Yáñez et.al found that it was feasible to produce (R)-3-hydroxybutyric acid from methane in vivo depolymerization of polyhydroxybutyrate in Methylocystis parvus OBBP [42]. Mai et.al also demonstrated that 2-hydroxyisobutyric acid could be produced from methane [43].

According to the results of our study, morpholine was found to be significantly associated with the enteric methane emissions in Angus. Shaukat et.al found morpholine was a developing solvent for CO_2_ capture, which had better reactivity with green gas such as CO_2_ [44]. In our study, phenylalanine was identified in KC and had a positive correlation with CH_4_ amount. Some previous researches have shown that the phenylalanine is a promoter in methane hydrate formation [45, 46].

**Fig 8:**
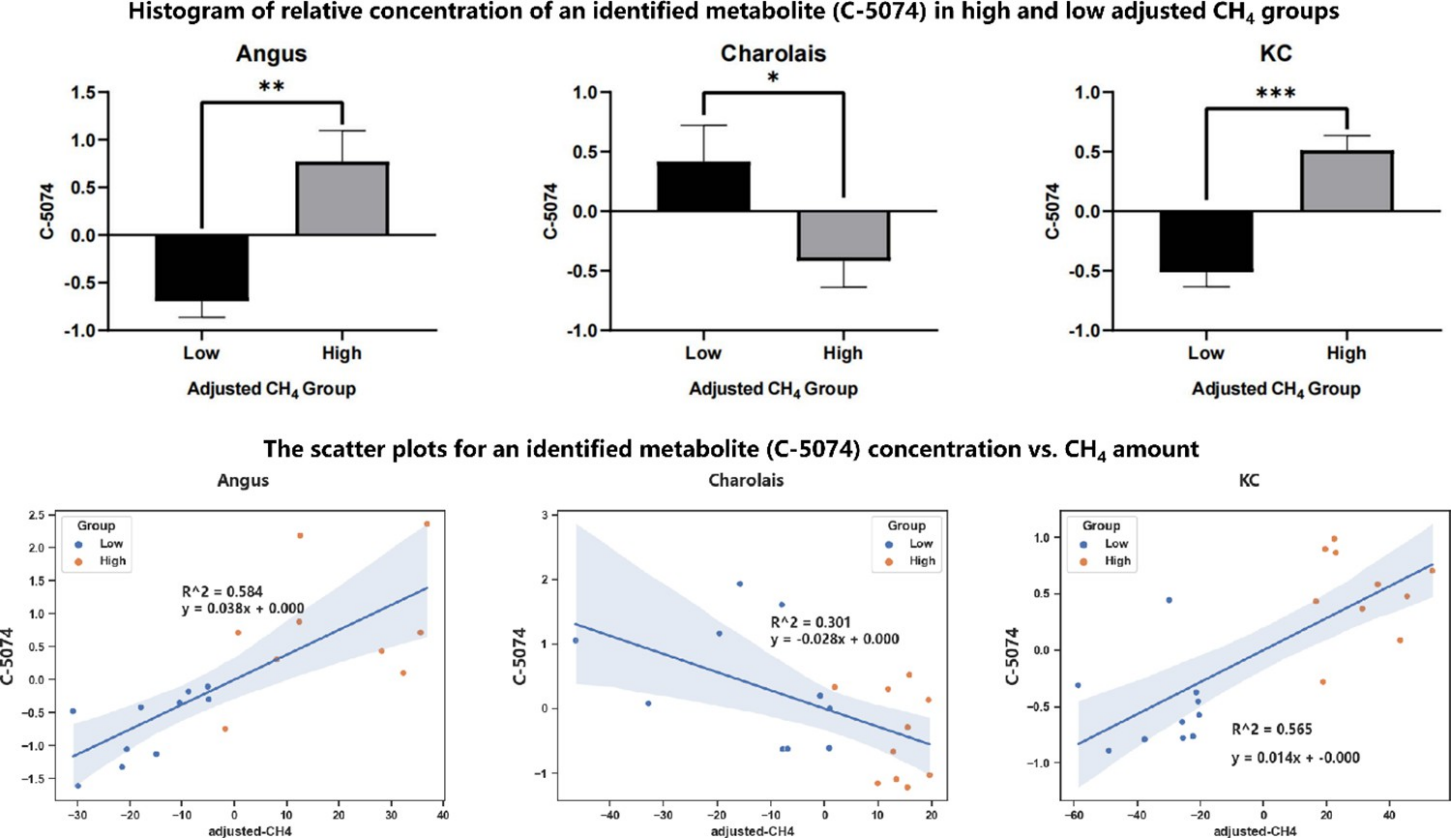
The histogram of relative concentrations of an identified metabolite (C-5074) in the high and low enteric methane emission (AVG_DAILYCH4) groups and its scatter plots of concentration vs. AVG_DAILYCH4 amount in the Angus, Charolais, and Kinsella Composite (KC) populations.

We also listed 44 common metabolites of Tier 3 in the three beef cattle populations detected by both the t-test and regression analysis in S7 Table. These statistically significant candidate and suggestive metabolites quantified using the 3-tier approach will also help identify metabolites as biomarkers to assist with selection of cattle with reduced methane emissions. For instance, C-5074 was one of the candidate metabolites detected by both the t-test and regression analyses in all three breed populations. The histogram of relative concentration of C-5074 in high and low adjusted CH_4_ group and the scatter plots for C-5074 concentration vs. CH_4_ amount in three populations was shown in Fig 8. Both of results of the t-test and regression analysis clearly indicated that the significant metabolite (C-5074) was positively associated with the amount of CH_4_ in both Angus and KC but negatively associated with the amount of CH_4_ in the Charolais population. Indeed, follow up studies are required to further characterize the metabolite and verify its association with methane emission through validation studies.

The metabolites detected in this study as associated with the methane emission will also help improve our understanding on the molecular basis of metabolite and methane emissions. With the help of pathway analysis, we can study and compare the importance of some pathways and metabolites, and this would facilitate our understanding of why different cows have different methane production and emission amounts.

For Angus breed (S2A Fig), the most affected pathways include “Arginine and proline metabolism”, “Arginine biosynthesis”, “Butanoate metabolism” and “Glutathione metabolism”. We took “Arginine and proline metabolism” as an example for more discussion (S2B Fig). L-ornithine is a non-essential, non-protein amino acid [47], and was significantly downregulated in high methane emission group. For Charolais breed (S3A Fig), “beta-Alanine metabolism”, “Pyruvate metabolism”, “Glycolysis / Gluconeogenesis” and “Citrate cycle (TCA cycle)” were the four most affected pathways, and as for the “beta-Alanine metabolism” (S3B Fig), we found a significant increase of 3-Oxopropanoate in high methane emission group.

For KC breed (S4A Fig), many pathways were affected and of those, “Phenylalanine, tyrosine and tryptophan biosynthesis”, “Phenylalanine metabolism”, “Arginine biosynthesis” and “Arginine and proline metabolism” were the four top affected ones. Here, we provide a discussion about the relation between methane emission and “Phenylalanine, tyrosine and tryptophan biosynthesis” as shown in S4B Fig. Phenylalanine is an aromatic essential amino acid. An increased concentration of phenylalanine in beef cattle with high methane emission amount was observed. A recent study also found the enrichment of phenylalanine in cows with higher milk production and less methanogen and methanogenesis functions, which may reveal the important role of the rumen microbiome [48].

Arginine and proline metabolism and arginine biosynthesis were two common pathways in all four top pathway detected by the both Angus and KC populations, which also supports the results that the Angus and KC populations had more common metabolites that were significantly associated with CH_4_. It was found that the alanine pathway was the center of interactions of many other pathways [49], which implied the activation of this pathway might be related to higher methane emissions. It is reported that Arginine and proline metabolism (detected in Angus and KC population) and Phenylalanine metabolism (detected in KC population) were significantly affected pathway on enteric methane emissions from dairy cows [50].

Furthermore, the above enriched pathways or molecular processes detected through analyzing metabolite samples of the Angus, Charolais, and KC populations with methane emissions will also help prioritize candidate genes to detect DNA polymorphisms that are associated with methane emissions in beef cattle.

## Conclusions

In this work, a high performance four-channel CIL LC-MS method was applied to profile amine/phenol -, carboxyl -, hydroxyl—and carbonyl submetabolomes of cow plasma samples. High metabolome coverage was achieved with the detection of 4235 metabolites in three tiers., T-tests, volcano plots, and regression analyses within breed population were conducted to detect metabolites that were significantly associated with the amount of methane emission. Our results showed that metabolites that were associated with methane emissions in beef cattle were largely breed-specific. The pathway analysis detected enriched metabolic processes related to methane production in beef cattle. The detected metabolites from the three beef cattle populations and enriched metabolic processes provide valuable resources to further characterize the metabolites and verify their associations with CH_4_ as biomarkers to assist in selection of cattle with reduced methane emissions.

## Supporting information

S1 Table The basic statistics of AVG_DAILYCH4 in different populations. (DOCX)

S2 Table The basic statistics of metabolites in different populations. (XLSX)

S3 Table The detailed metabolite detection and identification results. (XLSX)

S4 Table The metabolites identified by the t-test and regression analysis. (XLSX)

S5 Table The Log_2_FC, pvalues and directions for enteric methane emissions of each metabolite for the volcano plots in the Angus, Charolais, and Kinsella Composite (KC) populations. (XLSX)

S6 Table The pathway results with detailed pathway impact and p-value for the three breed populations. (XLSX)

S7 Table Common metabolites of Tier 3 detected to be associated with the enteric methane emissions (AVG_DAILYCH4) in the Angus, Charolais, and Kinsella Composite (KC) populations using both the t-test and regression analysis. (DOCX)

S1 Fig The normal QQ-plot for adjusted AVG_DAILYCH4 and adjusted relative metabolite concentrations (an example of A-5) in different populations. (TIF)

S2 Fig (A) Overview of metabolic pathway analysis for Angus breed. (B) Synthesis and degradation of ketone bodies.
(TIF)

S3 Fig (A) Overview of metabolic pathway analysis for Charolais breed. (B) Pathway of Alanine, aspartate and glutamate metabolism.
(TIF)

S4 Fig (A) Overview of metabolic pathway analysis for KC breed. (B) Pathway of Phenylalanine metabolism.
(TIF)

S5 Fig The sPLS-DA 2D score plot of Angus, Charolais and KC breed population for low enteric methane emission (AVG_DAILYCH4). (TIF)

S6 Fig The scatter plots for some uncommon identified metabolites concentration vs. CH_4_ amount. (TIF)

## References

[1] Davis M, Ahiduzzaman M, Kumar A. How will Canada’s greenhouse gas emissions change by 2050? A disaggregated analysis of past and future greenhouse gas emissions using bottom-up energy modelling and Sankey diagrams. Applied energy. 2018;220:754–86.

[2] Gerber PJ, Steinfeld H, Henderson B, Mottet A, Opio C, Dijkman J, et al. Tackling climate change through livestock: a global assessment of emissions and mitigation opportunities: Food and Agriculture Organization of the United Nations (FAO); 2013.

[3] Alexandratos N, Bruinsma J. World agriculture towards 2030/2050: the 2012 revision. 2012.

[4] Canada OE. National inventory report: greenhouse gas sources and sinks in Canada. 1990–2018. Available from: https://publications.gc.ca/site/eng/9.506002/publication.html.

[5] Johnson KA, Johnson DE. Methane emissions from cattle. Journal of animal science. 1995;73(8):2483–92. doi: 10.2527/1995.7382483x 8567486

[6] Williams C. Application of biological simulation models in estimating feed efficiency of finishing steers. Journal of animal science. 2010;88(7):2523–9. doi: 10.2527/jas.2009-2655 20348372

[7] Karisa B, Thomson J, Wang Z, Li C, Montanholi Y, Miller S, et al. Plasma metabolites associated with residual feed intake and other productivity performance traits in beef cattle. Livestock Science. 2014;165:200–11.

[8] Guo K, Li L. Differential 12C-/13C-isotope dansylation labeling and fast liquid chromatography/mass spectrometry for absolute and relative quantification of the metabolome. Anal Chem. 2009;81(10):3919–32. doi: 10.1021/ac900166a 19309105

[9] Han W, Sapkota S, Camicioli R, Dixon RA, Li L. Profiling novel metabolic biomarkers for Parkinson’s disease using in‐depth metabolomic analysis. Movement Disord. 2017;32(12):1720–8. doi: 10.1002/mds.27173 28880465 PMC5753769

[10] Huan T, Tran T, Zheng J, Sapkota S, MacDonald SW, Camicioli R, et al. Metabolomics analyses of saliva detect novel biomarkers of Alzheimer’s disease. Journal of Alzheimer’s Disease. 2018;65(4):1401–16. doi: 10.3233/JAD-180711 30175979

[11] Wu Y, Streijger F, Wang Y, Lin G, Christie S, Mac-Thiong J-M, et al. Parallel metabolomic profiling of cerebrospinal fluid and serum for identifying biomarkers of injury severity after acute human spinal cord injury. Scientific reports. 2016;6(1):1–14. 27966539 10.1038/srep38718 PMC5155264

[12] Buzatto AZ, Malkawi A, Sabi EM, Mujamammi AH, Li L, Abdel Rahman AM. Tissue Lipidomic Alterations Induced by Prolonged Dexamethasone Treatment. Journal of proteome research. 2021;20(3):1558–70. doi: 10.1021/acs.jproteome.0c00759 33557525

[13] Peng J, Guo K, Xia J, Zhou J, Yang J, Westaway D, et al. Development of isotope labeling liquid chromatography mass spectrometry for mouse urine metabolomics: quantitative metabolomic study of transgenic mice related to Alzheimer’s disease. Journal of proteome research. 2014;13(10):4457–69. doi: 10.1021/pr500828v 25164377

[14] Guo K, Li L. High-performance isotope labeling for profiling carboxylic acid-containing metabolites in biofluids by mass spectrometry. Anal Chem. 2010;82(21):8789–93. doi: 10.1021/ac102146g 20945833

[15] Zhao S, Luo X, Li L. Chemical isotope labeling LC-MS for high coverage and quantitative profiling of the hydroxyl submetabolome in metabolomics. Anal Chem. 2016;88(21):10617–23. doi: 10.1021/acs.analchem.6b02967 27690392

[16] Zhao S, Dawe M, Guo K, Li L. Development of High-Performance Chemical Isotope Labeling LC–MS for Profiling the Carbonyl Submetabolome. Anal Chem. 2017;89(12):6758–65. doi: 10.1021/acs.analchem.7b01098 28505421

[17] Zhao S, Li H, Han W, Chan W, Li L. Metabolomic coverage of chemical-group-submetabolome analysis: group classification and four-channel chemical isotope labeling LC-MS. Anal Chem. 2019;91(18):12108–15. doi: 10.1021/acs.analchem.9b03431 31441644

[18] Mao F, Chen L, Vinsky M, Okine E, Wang Z, Basarab J, et al. Phenotypic and genetic relationships of feed efficiency with growth performance, ultrasound, and carcass merit traits in Angus and Charolais steers. Journal of animal science. 2013;91. doi: 10.2527/jas.2012-5470 23463551

[19] Goonewardene LA, Wang Z, Price MA, Yang RC, Berg RT, Makarechian M. Effect of udder type and calving assistance on weaning traits of beef and dairy×beef calves. Livestock Production Science. 2003;81(1):47–56. doi: 10.1016/S0301-6226(02)00194-X

[20] Care CCoA. CCAC. CCAC guidelines on: the care and use of farm animals in research, teaching and testing. https://www.ccac.ca/Documents/Standards/Guidelines/Farm_Animals.pdf.: Canadian Council on Animal Care; 2009 [cited 2020 1 Feb].

[21] Manafiazar G, Zimmerman S, Basarab JA. Repeatability and variability of short-term spot measurement of methane and carbon dioxide emissions from beef cattle using GreenFeed emissions monitoring system. Canadian Journal of Animal Science. 2017;97(1):118–26. doi: 10.1139/cjas-2015-0190

[22] Wu Y, Li L. Determination of Total Concentration of Chemically Labeled Metabolites as a Means of Metabolome Sample Normalization and Sample Loading Optimization in Mass Spectrometry-Based Metabolomics. Anal Chem. 2012;84(24):10723–31. doi: 10.1021/ac3025625 23190334

[23] Zhou R, Tseng C-L, Huan T, Li L. IsoMS: automated processing of LC-MS data generated by a chemical isotope labeling metabolomics platform. Anal Chem. 2014;86(10):4675–9. doi: 10.1021/ac5009089 24766305

[24] Collingridge D. A Primer on Quantitized Data Analysis and Permutation Testing. Journal of Mixed Methods Research. 2013;7:81–97.

[25] Jin W, Riley RM, Wolfinger RD, White KP, Passador-Gurgel G, Gibson G. The contributions of sex, genotype and age to transcriptional variance in Drosophila melanogaster. Nat Genet. 2001;29(4):389–95. doi: 10.1038/ng766 . 11726925

[26] Cui X, Churchill GA. Statistical tests for differential expression in cDNA microarray experiments. Genome Biol. 2003;4(4):210. Epub 20030317. doi: 10.1186/gb-2003-4-4-210 ; PubMed Central PMCID: PMC154570. 12702200 PMC154570

[27] Lê Cao K-A, Rossouw D, Robert-Granié C, Besse P. A sparse PLS for variable selection when integrating omics data. Statistical applications in genetics and molecular biology. 2008;7(1). doi: 10.2202/1544-6115.1390 19049491

[28] Lê Cao K-A, Boitard S, Besse P. Sparse PLS discriminant analysis: biologically relevant feature selection and graphical displays for multiclass problems. BMC Bioinformatics. 2011;12(1):253. doi: 10.1186/1471-2105-12-253 21693065 PMC3133555

[29] Mukiibi R, Vinsky M, Keogh KA, Fitzsimmons C, Stothard P, Waters SM, et al. Transcriptome analyses reveal reduced hepatic lipid synthesis and accumulation in more feed efficient beef cattle. Scientific reports. 2018;8(1):7303. doi: 10.1038/s41598-018-25605-3 29740082 PMC5940658

[30] Mukiibi R, Vinsky M, Keogh K, Fitzsimmons C, Stothard P, Waters SM, et al. Liver transcriptome profiling of beef steers with divergent growth rate, feed intake, or metabolic body weight phenotypes1. Journal of animal science. 2019;97(11):4386–404. Epub 2019/10/05. doi: 10.1093/jas/skz315 ; PubMed Central PMCID: PMC6827404. 31583405 PMC6827404

[31] Li F, Hitch TCA, Chen Y, Creevey CJ, Guan LL. Comparative metagenomic and metatranscriptomic analyses reveal the breed effect on the rumen microbiome and its associations with feed efficiency in beef cattle. Microbiome. 2019;7(1):6. doi: 10.1186/s40168-019-0618-5 30642389 PMC6332916

[32] Paz HA, Anderson CL, Muller MJ, Kononoff PJ, Fernando SC. Rumen Bacterial Community Composition in Holstein and Jersey Cows Is Different under Same Dietary Condition and Is Not Affected by Sampling Method. Frontiers in microbiology. 2016;7:1206. Epub 2016/08/19. doi: 10.3389/fmicb.2016.01206 ; PubMed Central PMCID: PMC4971436. 27536291 PMC4971436

[33] Zhang Y, Li F, Chen Y, Guan LL. The Effects of Breed and Residual Feed Intake Divergence on the Abundance and Active Population of Rumen Microbiota in Beef Cattle. Animals: an open access journal from MDPI. 2022;12(15). Epub 2022/08/13. doi: 10.3390/ani12151966 ; PubMed Central PMCID: PMC9367312. 35953955 PMC9367312

[34] Briggs HM, editor Modern Breeds of Livestock1950.

[35] Crowley JJ, McGee M, Kenny DA, Crews DH Jr., Evans RD, Berry DP. Phenotypic and genetic parameters for different measures of feed efficiency in different breeds of Irish performance-tested beef bulls. Journal of animal science. 2010;88(3):885–94. Epub 2009/12/08. doi: 10.2527/jas.2009-1852 . 19966161

[36] Gregory KE, Cundiff LV, Koch RM, Dikeman ME, Koohmaraie M. Breed effects and retained heterosis for growth, carcass, and meat traits in advanced generations of composite populations of beef cattle. Journal of animal science. 1994;72(4):833–50. Epub 1994/04/01. doi: 10.2527/1994.724833x . 8014148

[37] MAYNARD LA LO OSLI JK. Animal Nutrition. Fourth Edition. Soil Science. 1956;82(3):259. 00010694-195609000-00009.

[38] Arthur P, Archer J, Richardson EC, Herd RM. Potential for selection to improve efficiency of feed use in beef cattle: A review. Australian Journal of Agricultural Research—AUST J AGR RES. 1999;50. doi: 10.1071/A98075

[39] Eng J. Sample size estimation: how many individuals should be studied? Radiology. 2003;227(2):309–13. Epub 2003/05/07. doi: 10.1148/radiol.2272012051 . 12732691

[40] Billoir E, Navratil V, Blaise BJ. Sample size calculation in metabolic phenotyping studies. Briefings in bioinformatics. 2015;16(5):813–9. doi: 10.1093/bib/bbu052 25600654

[41] Kim M, Masaki T, Ikuta K, Iwamoto E, Nishihara K, Hirai M, et al. Physiological responses and adaptations to high methane production in Japanese Black cattle. Scientific reports. 2022;12(1):11154. Epub 2022/07/02. doi: 10.1038/s41598-022-15146-1 ; PubMed Central PMCID: PMC9249741. 35778422 PMC9249741

[42] Yáñez L, Rodríguez Y, Scott F, Vergara-Fernández A, Muñoz R. Production of (R)-3-hydroxybutyric acid from methane by in vivo depolymerization of polyhydroxybutyrate in Methylocystis parvus OBBP. Bioresource technology. 2022;353:127141. Epub 2022/04/12. doi: 10.1016/j.biortech.2022.127141 . 35405209

[43] Mai DHA, Nguyen TT, Lee EY. The ethylmalonyl-CoA pathway for methane-based biorefineries: a case study of using Methylosinus trichosporium OB3b, an alpha-proteobacterial methanotroph, for producing 2-hydroxyisobutyric acid and 1,3-butanediol from methane. Green Chemistry. 2021;23(19):7712–23. doi: 10.1039/D1GC02866A

[44] Mazari SA, Abro R, Bhutto AW, Saeed IM, Ali BS, Jan BM, et al. Thermal degradation kinetics of morpholine for carbon dioxide capture. Journal of Environmental Chemical Engineering. 2020;8(3):103814. doi: 10.1016/j.jece.2020.103814

[45] Liu Y, Chen B, Chen Y, Zhang S, Guo W, Cai Y, et al. Methane Storage in a Hydrated Form as Promoted by Leucines for Possible Application to Natural Gas Transportation and Storage. Energy Technology. 2015;3. doi: 10.1002/ente.201500048

[46] Veluswamy H, Hong Q, Linga P. Morphology Study of Methane Hydrate Formation and Dissociation in the Presence of Amino Acid. Crystal Growth & Design. 2016;16:5932–45. doi: 10.1021/acs.cgd.6b00997

[47] Miyake M, Kirisako T, Kokubo T, Miura Y, Morishita K, Okamura H, et al. Randomised controlled trial of the effects of L-ornithine on stress markers and sleep quality in healthy workers. Nutrition journal. 2014;13:53. Epub 2014/06/04. doi: 10.1186/1475-2891-13-53 ; PubMed Central PMCID: PMC4055948. 24889392 PMC4055948

[48] Xue M-Y, Sun H-Z, Wu X-H, Liu J-X, Guan LL. Multi-omics reveals that the rumen microbiome and its metabolome together with the host metabolome contribute to individualized dairy cow performance. Microbiome. 2020;8:1–19. 32398126 10.1186/s40168-020-00819-8 PMC7218573

[49] López-Diez L, Calle-Velásquez C, Hanigan MD, Ruiz-Cortés ZT. Amino Acid Metabolomic Profiles in Bovine Mammary Epithelial Cells under Essential Amino Acid Restriction. Animals. 2021;11(5):1334. doi: 10.3390/ani11051334 34067229 PMC8151660

[50] Yanibada B, Ulli M, Pétéra M, Canlet C, Durand S, Jourdan F, et al. Milk metabolome reveals variations on enteric methane emissions from dairy cows fed a specific inhibitor of the methanogenesis pathway. Journal of dairy science. 2021;104. doi: 10.3168/jds.2021-20477 34531049

