# Supplementary material for: Analyses of plasma metabolites using a high performance four-channel CIL LC-MS method and identification of metabolites associated with enteric methane emissions in beef cattle
Source: PLoS One. 2024 Mar 1;19(3):e0299268. doi: 10.1371/journal.pone.0299268 (PMC10906882; doi:10.1371/journal.pone.0299268)
Supplement: S1 Table — (DOCX) [file pone.0299268.s001.docx]

**S1 Table. The basic statistics of AVG_DAILYCH4 in different populations**

| **Population** | **Number of records** | **Mean±se** | **Max** | **Min** |
| --- | --- | --- | --- | --- |
| Angus | 19 | 170.72±5.59 | 207.32 | 133.81 |
| Charolais | 20 | 168.94±4.45 | 195.33 | 125.92 |
| KC | 20 | 243.74±7.72 | 289.97 | 183.80 |
