# Supplementary material for: Analyses of plasma metabolites using a high performance four-channel CIL LC-MS method and identification of metabolites associated with enteric methane emissions in beef cattle
Source: PLoS One. 2024 Mar 1;19(3):e0299268. doi: 10.1371/journal.pone.0299268 (PMC10906882; doi:10.1371/journal.pone.0299268)
Supplement: S7 Table — (DOCX) [file pone.0299268.s007.docx]

**S6 Table. Common metabolites of Tier 3 detected to be associated with the enteric methane emissions (AVG_DAILYCH4) in the Angus, Charolais, and Kinsella Composite (KC) populations using both the t-test and regression analysis**

| **Metabolite ID** | **Identification Level** | **Angus** | **Charolais** | **KC** |
| --- | --- | --- | --- | --- |
| C-6906 | Tier 3 zero-reaction | + | - | + |
| C-6245 | Tier 3 zero-reaction | + | - | + |
| C-5074 | Tier 3 one-reaction | + | - | + |
| C-5231 | Tier 3 one-reaction | + | - | + |
| C-5566 | Unidentified | + | - | + |
| H-526 | Unidentified | - | + | - |
| C-6480 | Tier 3 zero-reaction | + | - | + |
| C-6339 | Tier 3 zero-reaction | + | - | + |
| C-6977 | Tier 3 zero-reaction | + | - | + |
| C-1854 | Unidentified | + | - | + |
| C-6979 | Tier 3 one-reaction | + | - | + |
| C-5483 | Tier 3 zero-reaction | + | - | + |
| C-6229 | Tier 3 one-reaction | + | - | + |
| C-6227 | Tier 3 zero-reaction | + | - | + |
| C-5371 | Tier 3 one-reaction | + | - | + |
| C-2963 | Tier 3 zero-reaction | + | - | + |
| C-3359 | Tier 3 zero-reaction | + | - | + |
| C-6634 | Tier 3 zero-reaction | + | - | + |
| C-5720 | Tier 3 zero-reaction | + | - | + |
| C-5065 | Tier 3 one-reaction | + | - | + |
| C-4109 | Tier 3 zero-reaction | + | - | + |
| C-5479 | Tier 3 one-reaction | + | - | + |
| C-3577 | Tier 3 only two-reaction | + | - | + |
| K-3191 | Tier 3 only two-reaction | - | + | - |
| C-5414 | Tier 3 zero-reaction | + | - | + |
| C-3811 | Tier 3 zero-reaction | + | - | + |
| C-6598 | Tier 3 zero-reaction | + | - | + |
| C-3059 | Tier 3 zero-reaction | + | - | + |
| H-2046 | Tier 3 zero-reaction | - | + | - |
| C-3899 | Tier 3 zero-reaction | + | - | + |
| C-5182 | Tier 3 zero-reaction | + | - | + |
| C-3560 | Tier 3 zero-reaction | + | - | + |
| C-3420 | Tier 3 zero-reaction | + | - | + |
| A-1828 | Tier 3 only two-reaction | - | + | - |
| C-4406 | Tier 3 zero-reaction | + | - | + |
| C-4401 | Tier 3 zero-reaction | + | - | + |
| C-5001 | Tier 3 zero-reaction | + | - | + |
| C-4693 | Unidentified | + | - | + |
| C-3773 | Tier 3 zero-reaction | + | - | + |
| C-3830 | Tier 3 zero-reaction | + | - | + |
| C-2431 | Tier 3 zero-reaction | + | - | + |
| A-1785 | Tier 3 one-reaction | + | - | + |
| C-4493 | Tier 3 zero-reaction | + | - | + |
| C-4739 | Tier 3 zero-reaction | + | - | + |

+: The metabolite is positively associated with amount of CH_4_

-: The metabolite is negatively associated with amount of CH_4_
