# Supplementary figures and images for: Analyses of plasma metabolites using a high performance four-channel CIL LC-MS method and identification of metabolites associated with enteric methane emissions in beef cattle
Source: PLoS One. 2024 Mar 1;19(3):e0299268. doi: 10.1371/journal.pone.0299268 (PMC10906882; doi:10.1371/journal.pone.0299268)

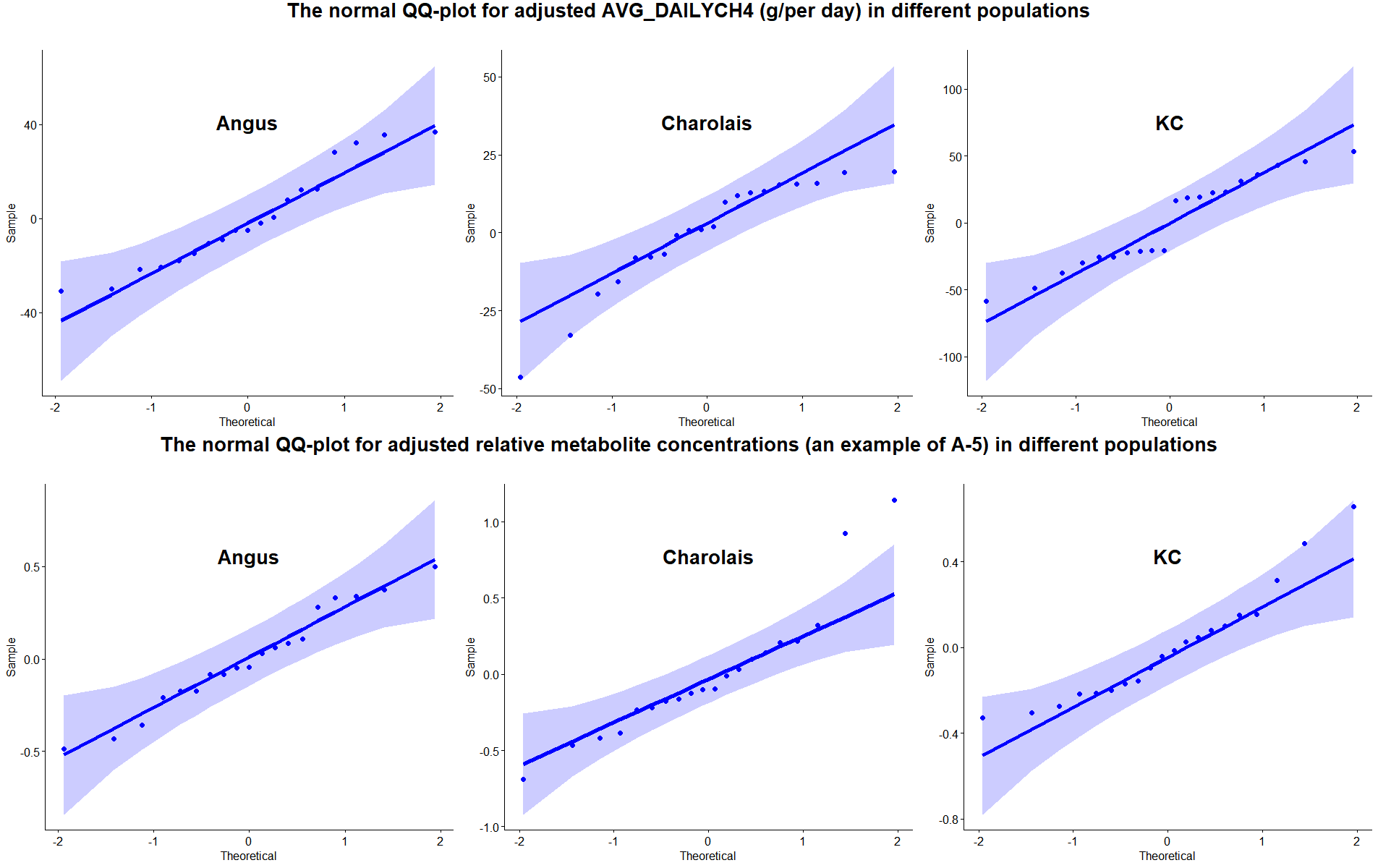

Supplement: S1 Fig — (TIF) [file pone.0299268.s008.tif]

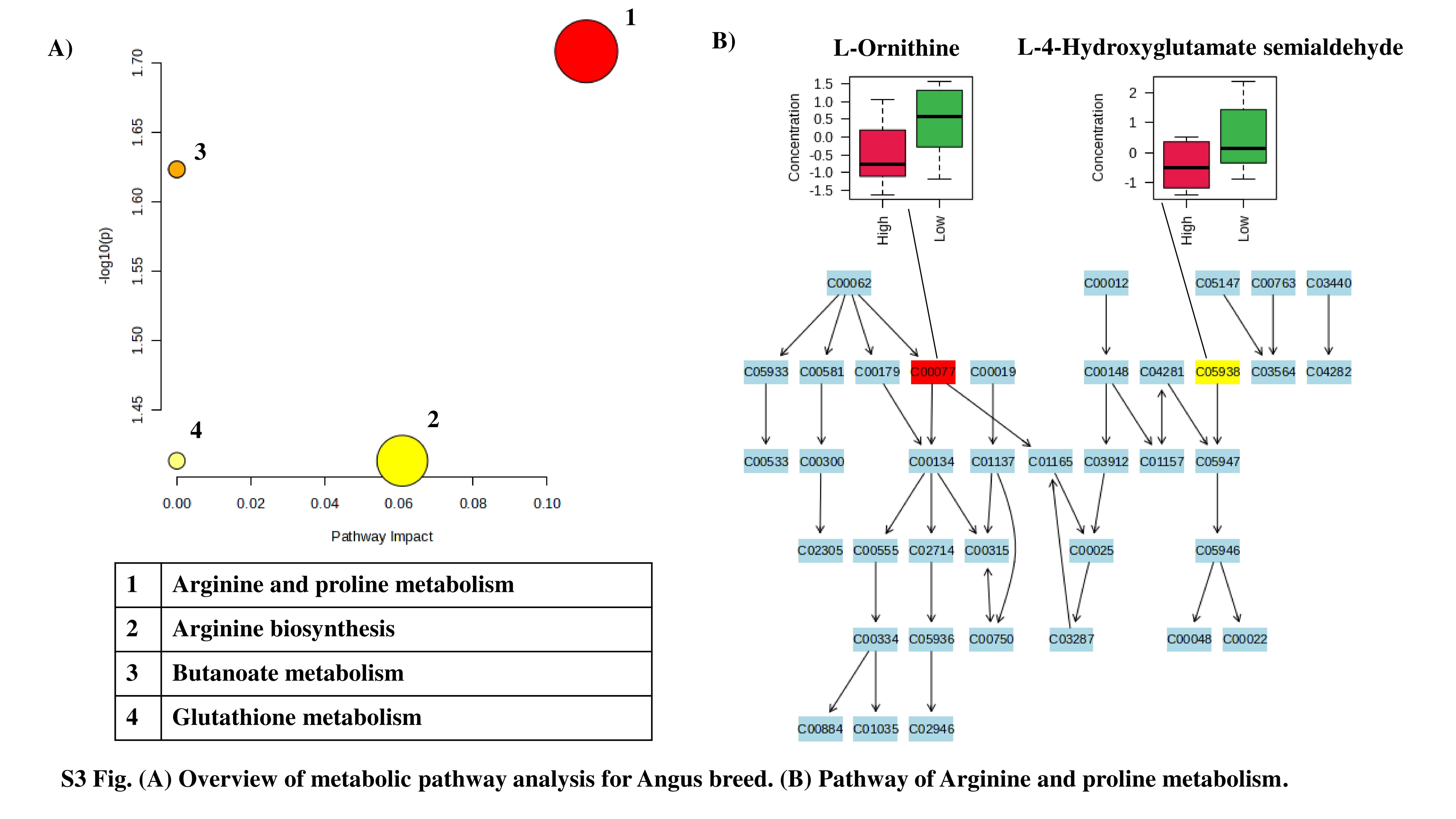

Supplement: S2 Fig — (A) Overview of metabolic pathway analysis for Angus breed. (B) Synthesis and degradation of ketone bodies. (TIF) [file pone.0299268.s009.tif]

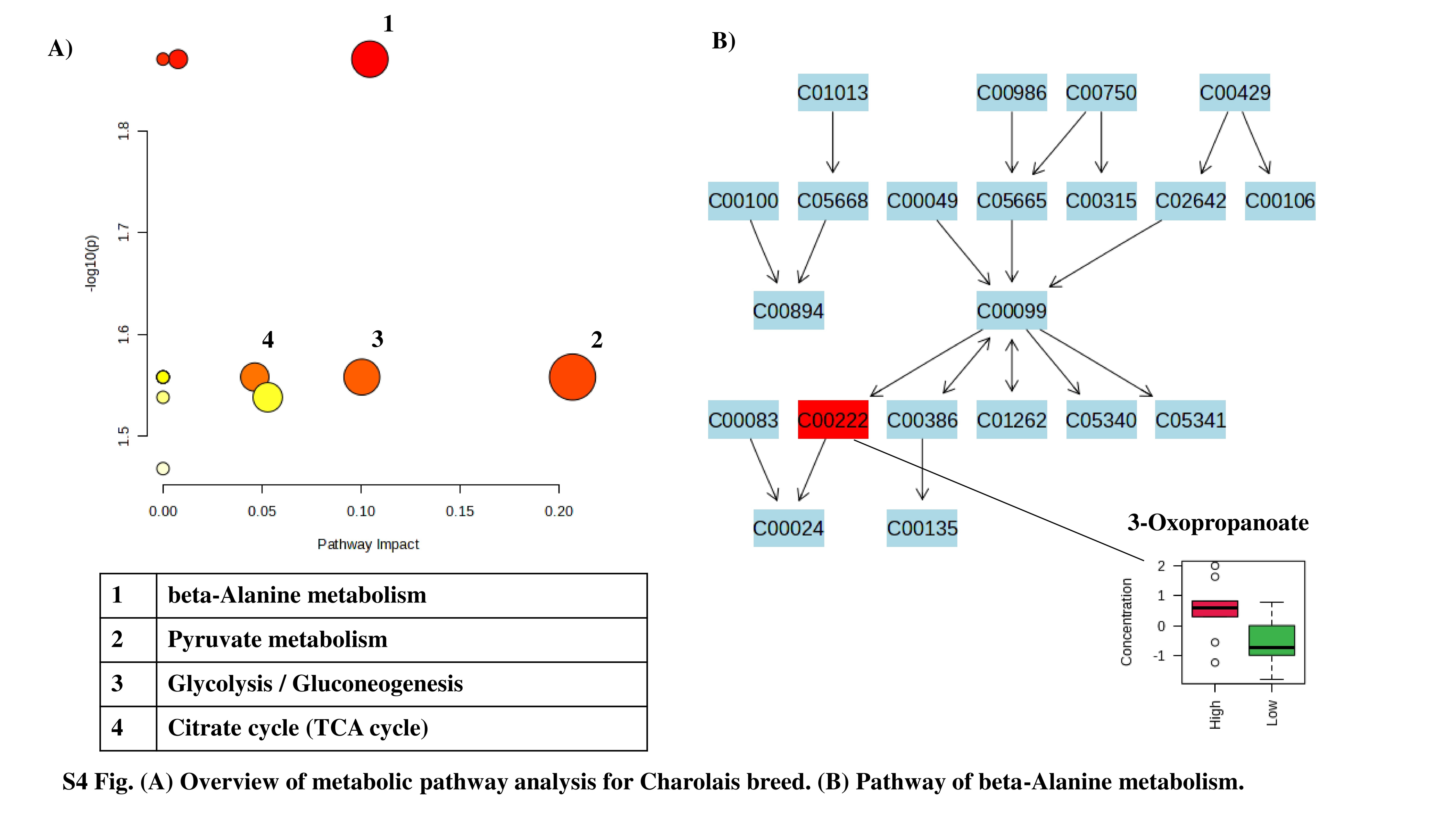

Supplement: S3 Fig — (A) Overview of metabolic pathway analysis for Charolais breed. (B) Pathway of Alanine, aspartate and glutamate metabolism. (TIF) [file pone.0299268.s010.tif]

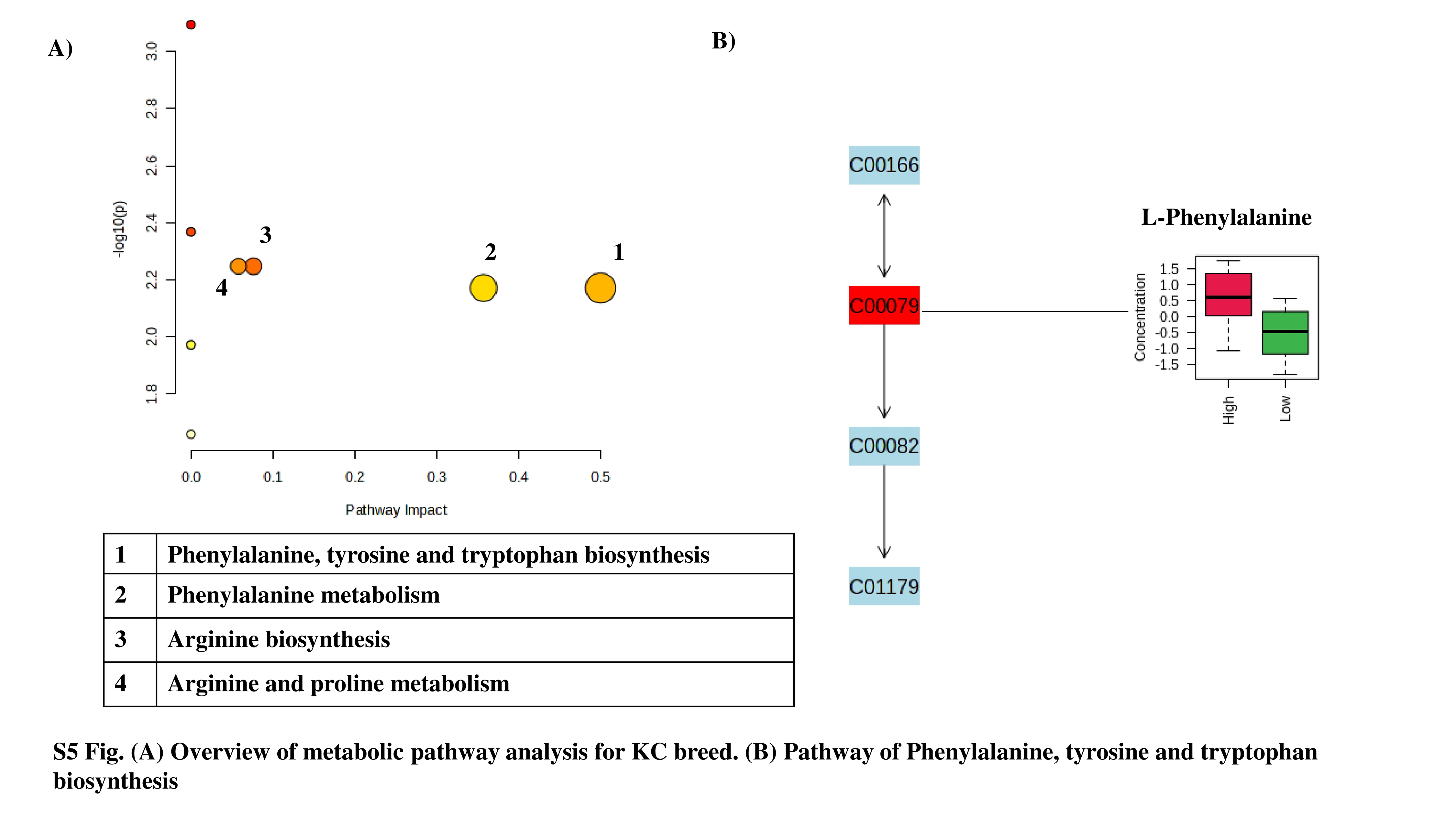

Supplement: S4 Fig — (A) Overview of metabolic pathway analysis for KC breed. (B) Pathway of Phenylalanine metabolism. (TIF) [file pone.0299268.s011.tif]

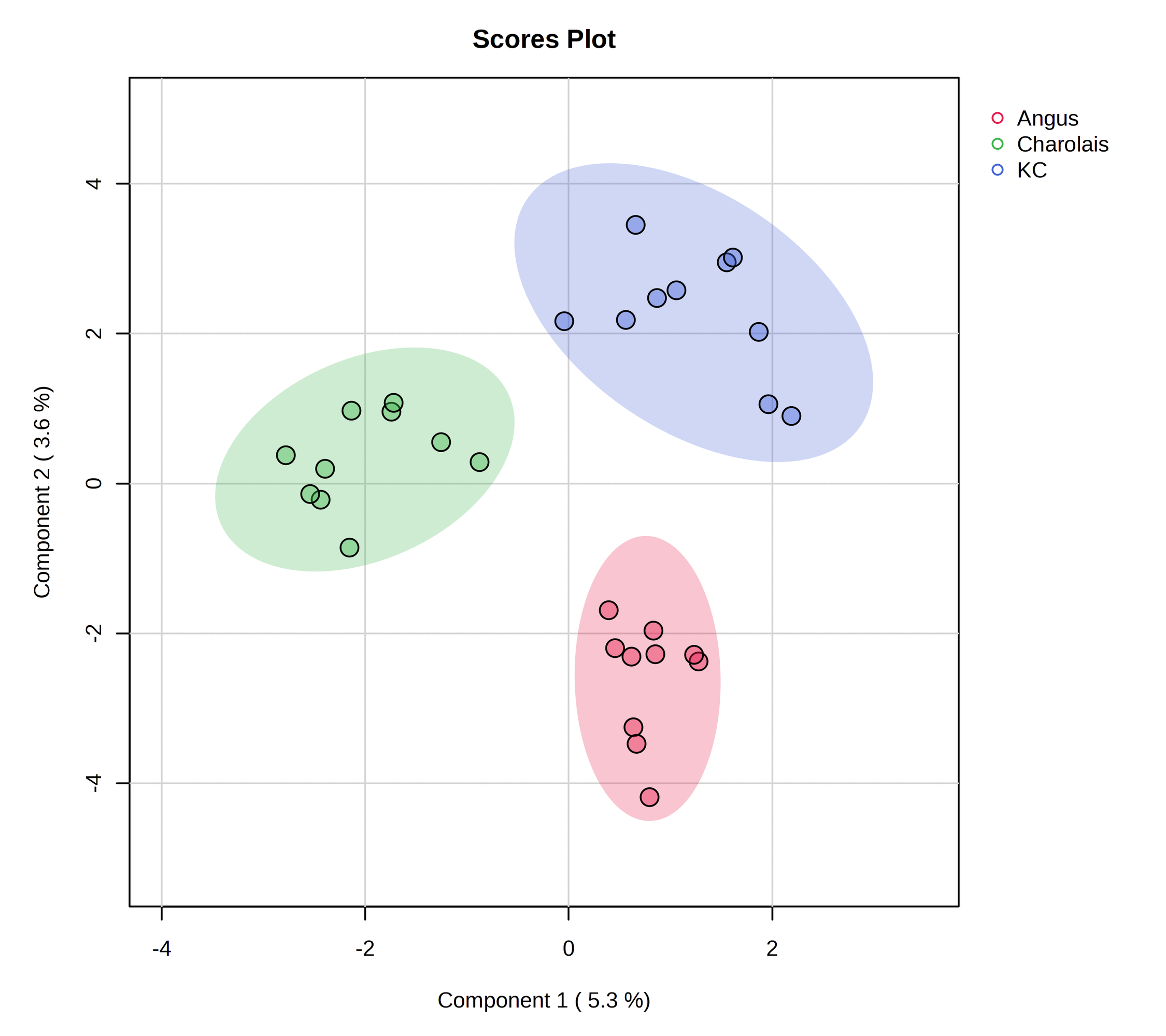

Supplement: S5 Fig — (TIF) [file pone.0299268.s012.tif]

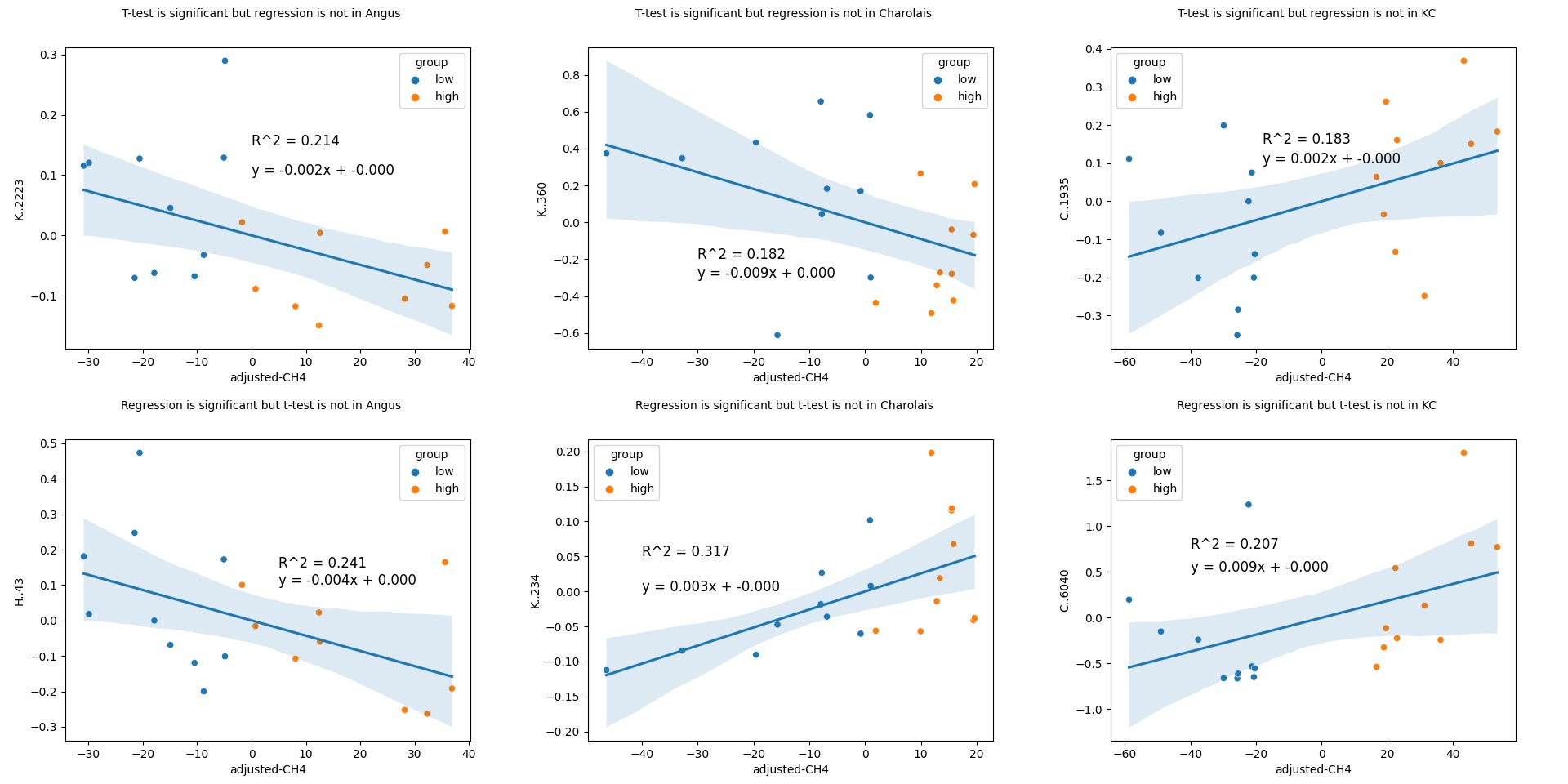

Supplement: S6 Fig — (TIF) [file pone.0299268.s013.tif]
